# Clustering and Principal Component Analysis as Distinct Computational Regimes of a Biologically Motivated Neural Circuit

**DOI:** 10.3390/brainsci16090930

**Published:** 2026-08-31

**Authors:** Chang Liu, Elijah F. W. Bowen, Richard Granger

**Affiliations:** 1 Thayer School of Engineering, Dartmouth College, Hanover, NH 03755, USA; charles.liu.th@dartmouth.edu; 2 Department of Psychological and Brain Sciences, Dartmouth College, Hanover, NH 03755, USA; elijah.floyd.william.bowen@dartmouth.edu

**Keywords:** thalamocortical circuits, lateral inhibition, component analysis, clustering, machine learning

## Abstract

**Background:** Characteristic circuitry in superficial layers of neocortex has been repeatedly shown to carry out lateral inhibition: excitatory–inhibitory interactions within a specific anatomical design. **Methods:** We provide simulation and formal analyses of the emergent operations of this circuitry. **Results:** Derived directly from anatomical circuit layout and physiological activation patterns, we show that this circuit carries out two distinct effective procedures on its inputs: categorization, and component analysis; moreover, we show that each procedure’s emergence is dependent on a single biological parameter: the relative strength of local feedback inhibitory cells. We characterize the detailed nature of both the biological activity and the emergent statistical operations and evaluate them in the context of extensive related literature in statistics, machine learning, and computational neuroscience. **Conclusions:** Very notably, the two emergent operations (clustering and component analysis) have not previously been shown to contain deep mathematical connections to each other, let alone to each be derivable from a single overarching algorithmic precursor that has clustering and component analysis as two special cases. The identification of that deep formal mathematical connection, and its arrival directly from a detailed biological circuit, represents a rare instance of novel mathematical relations arising from biological analyses.

## 1. Key Characteristics of Superficial Cortical Circuitry Produce Specific Algorithms

We begin by considering a central question: to what extent can canonical statistical computations be associated with the structure and dynamics of cortical microcircuits? Rather than treating neural circuits as generic substrates for arbitrary learning rules, we adopt a bottom-up perspective in which specific anatomical and physiological motifs may give rise to well-defined computational operations. In particular, we focus on local excitatory–inhibitory assemblies in superficial cortex and examine how their connectivity and temporal dynamics can support distinct regimes of information processing. As discussed in the following sections, variations in the strength and role of feedback inhibition are associated with qualitatively different behaviors—ranging from competitive, winner-take-all dynamics to cooperative, population-level representations. These regimes will later be related to well-studied statistical operations, suggesting a bridge between biological circuitry and algorithmic function.

### 1.1. Bottom-Up Biological Derivation of Circuit Components and Assemblies

**Figure 1:**
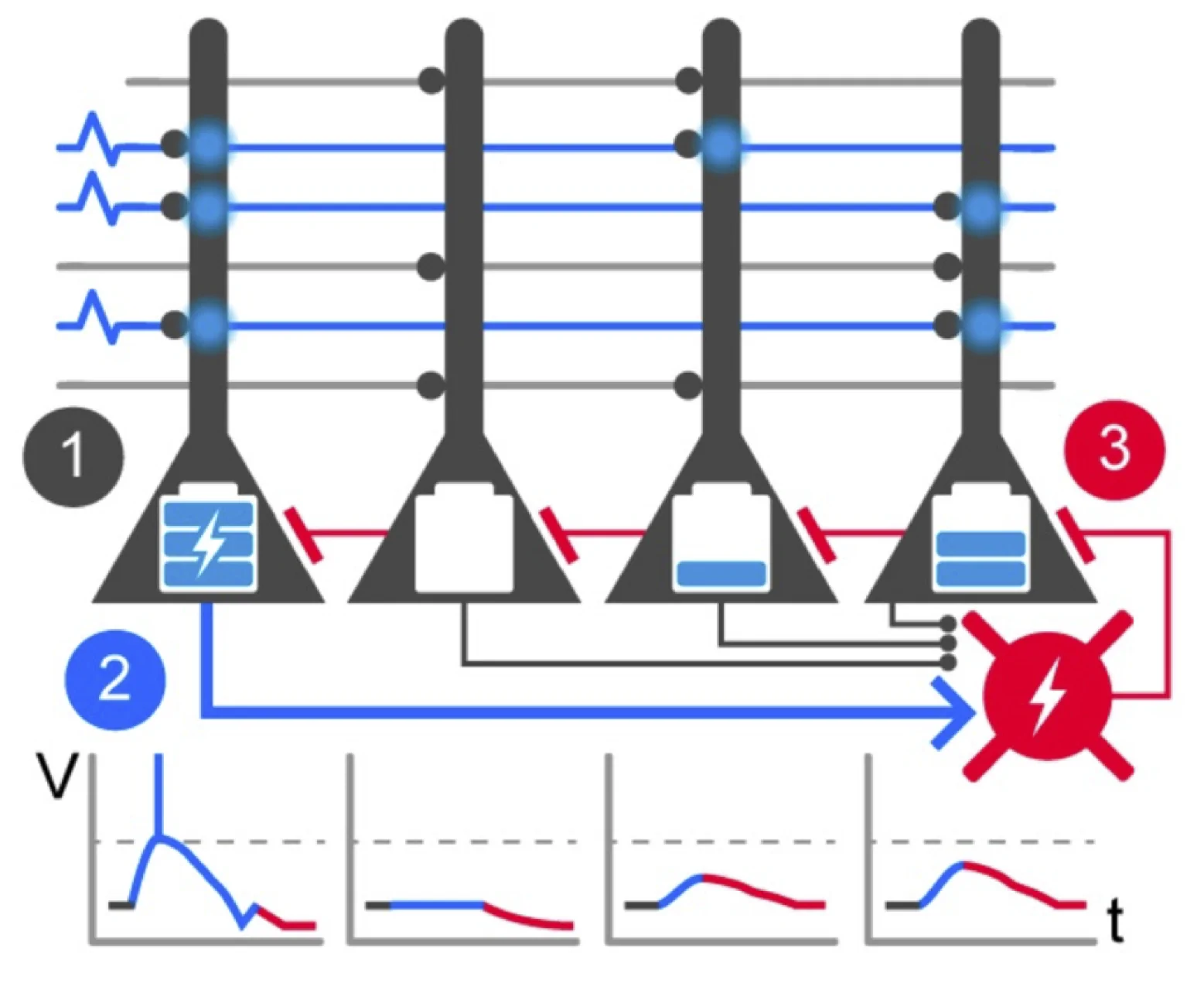
A cortical assembly composed of one inhibitory (red) and four excitatory (black) cells. Time-dependent voltage transients within the cells, shown as voltage (V) vs. time (t) graphs, build up over time as a function of the number of active input axons, and the number and weights of connected target dendritic synapses. The overall effect (see text) is that the most-activated excitatory cell (“1” in the figure) reaches its action potential threshold more rapidly than other cells; once any excitatory cell spikes, it activates its local inhibitory cell (2), which in turn inhibits all local excitatory cells (3). Thus, only the most-activated excitatory cell(s) can respond (i.e., in a sense, the “winners” take all).

Most cortical regions incorporate superficial layer cell assemblies consisting of excitatory and inhibitory cells anatomically organized as in Figure 1. Evidence suggests that these local excitatory–inhibitory assemblies contain both sparse external cortico-cortical input axons, and dense reciprocal local connectivity between excitatory and local inhibitory cells [1,2,3,4,5,6].

We propose a model of these assemblies that attempts to closely match both their anatomical layout and the nature and timing of their physiological interactions; we term these as local-feedback lateral inhibition circuits (L-FLICs).

These L-FLIC assemblies are connected such that (1) each glutamatergic input axon from other cortical cells, makes contact sparsely with a fixed number of excitatory target dendrites and (2) each excitatory dendrite receives contacts from a separately fixed number of incoming axons [7]. Furthermore, (3) the excitatory cells densely contact local inhibitory cells (in a ratio of about 4:1) and (4) the inhibitory cells reciprocally make dense contacts with local excitatory cells [8,9,10].

Within a local L-FLIC assembly, when an activated excitatory neuron rises to spiking threshold, it activates its local inhibitory neuron, which then inhibits all other excitatory neurons within the radius of its local axonal arborization (which includes the excitatory cell that activated the inhibitory cell). Once an input pattern arrives spatially distributed across incoming axons, then all target excitatory neurons’ voltages rise, until the most rapidly rising cells attain their spiking threshold, at which point the emitted spikes activate the local inhibition, which inhibits all the local excitatory cells within its reach. This will halt the rise of voltage in the excitatory cells, so that any that have not yet spiked in response to the input, now will be unable to do so. Thus, under this proposed model, only a limited subset of target excitatory cells is expected to respond to a given input.

The cells that do respond to a given input are determined by the speed of their voltage rise to their spiking threshold, which in turn is determined by the “fit” between the spatial pattern of incoming axon activity, and the distribution and conductivity of the sparsely distributed synapses on the dendrites of the target excitatory cells. Those cells with sufficiently conductive synapses that are connected to incoming axons that are active for this given input, are the cells that are likely to reach spiking threshold in response to this input.

### 1.2. Competitive Circuit Activity

The detailed unfolding of neuronal activity in these assemblies begins with an incoming set of spikes from axons arriving from some projecting cortical area. These in turn induce depolarization via input axons that contact dendritic synapses of neurons within the L-FLIC assemblies in the target cortical area. The total voltage over time in the target cells rises at a rate that is dependent on the quantity of input activation at a given target cells and the conductivity or weights of the receiving synapses (these are depicted as voltage/time graphs in Figure 1).

Note also that the active synapses of the target cells will then become increased slightly in weight, due to synaptic plasticity. These synaptic increases then have an effect on any future encounters with this input pattern or with slight variants of the same input pattern. If synapses have increased their weights in response to a spatial input activation pattern *A*, then in response to slightly different pattern A′, many of the strengthened synapses will be activated (due to the similarity between *A* and A′), and that will generate a more rapid voltage rise in the target cell, making that cell more likely to reach its spiking threshold before being halted by feedback lateral inhibition. In other words, for any input that elicits some target cell spiking, variants of that input will also tend to elicit spikes from the same target cells, and synaptic plasticity further increases this probability of the same target cells responding to similar but somewhat-different inputs. As a result, similar inputs will increasingly come to elicit identical, not just similar, cortical responses.

Such an operation bears a close resemblance to clustering, a core unsupervised learning technique that partitions data into groups based on similarity. In clustering, data points are grouped into clusters such that those within the same cluster exhibit stronger similarities—such as higher inclination, shorter distances, or more alike features—compared to points in different clusters.

A set of information-processing operations is thus carried out directly from the anatomical organization and physiological time-sequence activity of a small assembly of well-studied excitatory and inhibitory cells. These assemblies are very distinct from standard artificial-neural-network (ANN) approaches to cell operation (e.g., [11,12,13,14,15,16,17]). Many of these ANN approaches can be characterized in terms of “winner take all” or “lateral inhibition”, and they bear some notable points of correspondence with the L-FLIC model, as well as several important differences, that will be discussed.

### 1.3. Cooperative Circuit Activity

Ascending neuromodulatory systems, including the norepinephrine system originating from the locus coeruleus, the acetylcholine system from the basal forebrain, and the dopamine systems from the ventral tegmental area and substantia nigra pars compacta (SNc), are known to substantially modulate cortical activity [18]. These systems act on both pyramidal cells and cortical interneurons, and experimental studies have demonstrated that their modulatory effects can include suppression of inhibitory interneuron activity and, consequently, transient disinhibition of cortical excitatory populations [19,20,21,22,23,24,25,26,27,28,29]. These experimentally observed modulatory and disinhibitory mechanisms provide the biological motivation for considering changes in the effective strength of lateral inhibition in the L-FLIC framework. Importantly, however, the specific weak-inhibition regime analyzed below is a computational hypothesis motivated by these observations, rather than a direct experimentally established mapping between a particular ascending neuromodulatory system and the L-FLIC circuit.

We therefore ask what computational behavior may emerge in the L-FLIC circuit when lateral feedback inhibition is substantially weakened. In this hypothesized operating regime, excitatory activity still recruits the local feedback inhibitory cell, but the effective inhibitory influence on neighboring excitatory cells is assumed to be damped or suppressed. Consequently, multiple excitatory cells may reach their spiking thresholds, in contrast with the relatively few (or single) excitatory responses expected under strong lateral inhibition. As before, excitatory cells that respond sufficiently strongly are assumed to undergo activity-dependent synaptic strengthening at the synapses receiving the activating inputs. Over repeated trials, this produces a population of excitatory cells whose learned responses reflect the structure of the input. The following sections investigate, mathematically and computationally, what information such a population may represent under this weak-inhibition assumption.

Intuitively, each cell can be considered as a synaptic vector, and synaptic change causes that vector to come to “point” more closely in the direction of the input activity vectors on which the responding cells were trained. Thus all of these excitatory cells will change direction as a function of the inputs they respond to. Which inputs do they respond to? In this case, almost all of them, since the local inhibitory cell has been weakened and cannot provide lateral inhibition that would select only certain responding excitatory cells. If the excitatory cells are thus being “trained” to respond to the population of inputs, these cells will tend to point in the general direction of the overall leading principal component of these inputs. This line of intuitive reasoning is subjected to formal treatment in subsequent sections. The subsequent mathematical analysis suggests that the learned response can be viewed as belonging to a broader family of principal-component-like responses. We refer to this computational response as “Antipodal Iterative Mean Estimation (AIME)”, which is analyzed mathematically in the following sections.

### 1.4. Partial Summary

In sum, (i) there is a local excitatory–inhibitory circuit architecture that occurs widely throughout superficial layers of cortex; (ii) the parameters of the activities of neurons within this architecture can be adjusted, biologically, by “modulatory” input from other brain systems (such as “ascending systems”), which can regulate the strength of inhibitory cells in this architecture; (iii) the circuit’s characteristic responses to structured (non-uniform) populations of inputs can then be formally characterized, both in the high-feedback-inhibition, and low-feedback-inhibition cases, depending on the modulation of the inhibitory cells. Analysis of the proposed model suggests that these two parameterized modes of operation exhibit computational behaviors that are closely related to well-studied statistical operations, namely clustering and component analysis. The computational characteristics of these two regimes are analyzed in detail in the following sections.

The competitive operating regime of the L-FLIC circuit, in which local feedback inhibition remains strong, forms the basis of the computational framework previously proposed by Rodriguez et al. [30]. As reviewed above, extensive experimental studies have shown that ascending neuromodulatory systems can transiently suppress cortical inhibitory activity. This raises a biologically motivated question: how would the same circuit behave if its local feedback inhibition were substantially weakened? In the present work, we investigate this cooperative operating regime as a computational hypothesis derived from an experimentally plausible physiological mechanism. The following sections characterize the computational behavior that emerges under these conditions through mathematical analysis and simulation. If feedback inhibition is weak or absent, the initially responding cells will still activate the local feedback inhibitory cell; however, the inhibition is now damped or suppressed by the ascending input, resulting in substantially reduced lateral feedback inhibition of local excitatory cells. Consequently, multiple excitatory cells may reach their spiking thresholds, in contrast to the competitive regime in which only relatively few (or even a single) excitatory cells respond.

Clustering and component analysis (e.g., Principal Component Analysis) are typically studied as distinct statistical operations. While prior work has explored connections between them—through vector space formulations (e.g., [31,32,33,34,35]) or by integrating elements of one method into the procedures of the other [36,37,38,39,40,41]—these approaches generally maintain a separation between the two paradigms at the algorithmic level. We provide a more detailed discussion of the related work in Section 7 of this paper.

In this work, we investigate a single biologically motivated circuit architecture whose operating regimes exhibit computational behaviors resembling clustering and component-analysis-like processing. Specifically, the operating regime of the circuit is modulated by the activity of the feedback inhibitory population. Under strong inhibition, the resulting behavior is consistent with competitive clustering-like computation, whereas under weak or absent inhibition, the resulting behavior resembles component-analysis-like processing. Both regimes are characterized and analyzed in detail within this framework.

Taken together, these observations suggest that a common circuit architecture can support multiple modes of computation under different operating conditions. Rather than establishing a formal algebraic equivalence between classical statistical algorithms, our goal is to highlight a conceptual and algorithmic convergence: the same biological circuit motif can exhibit computational behaviors closely aligned with both clustering and component analysis under different parameter regimes.

Notably, this relationship emerges from a biologically derived model, suggesting that a single neural circuit motif that is widespread in actual brains, may support multiple canonical statistical functions depending on its operating regime. While this connection is not expressed as a single closed-form derivation, it points toward a possible organizing principle linking statistical computation with neural architecture.

Finally, we position this work within a brain-inspired engineering framework. The circuit model studied here is a hypothesized construct and has not yet been directly validated through biological experimental measurements. Nevertheless, its design is grounded in biologically plausible motifs drawn from the neuroscience literature, and its behavior is supported by explicit mathematical formulation and computational simulation. Accordingly, we view the primary contribution of this work not as establishing a new algebraic relationship between existing statistical methods, but as introducing a biologically motivated computational framework whose behavior can be analyzed mathematically and compared with established statistical algorithms. From this perspective, the framework generates concrete, testable hypotheses regarding how different computational behaviors may emerge from shared neural circuitry, thereby providing a basis for future theoretical and experimental investigation.

The remainder of this paper is structured as follows. In Section 2, we present an overview of the L-FLIC’s clustering algorithm (under the competitive dynamics), along with its downstream thalamocortical circuits responsible for hierarchical clustering. As this algorithm has been fully validated in the work of Rodriguez et al. [30], we briefly summarize its computational outcomes in this paper, treating it as a demonstration of the “competitive circuits activity” readily adopted from prior work. In contrast, Section 3, Section 4 and Section 5 are devoted to the development of the “cooperative circuit activity” model, referred to as the AIME algorithm. We intentionally place greater emphasis on the mathematical formulation, comparison with standard methods (PCA), and analysis of computational results in the context of AIME. In the last two sections, we review the influential work related to clustering of such kinds, biological-PCA methods, and PCA-clustering connections, which, while not directly foundational to our derivations of AIME, provide useful context for situating our contributions. Our findings provide a computational perspective suggesting how clustering-like and component-analysis-like behaviors may arise from different operating regimes of the same L-FLIC architecture, offering conceptual and methodological insights within a biologically motivated computational framework.

## 2. Competitive Circuit Activity—Clustering

The cells thus far described in cortical superficial layers’ L-FLIC are combined with several additional layers of cells in neocortical circuitry (layers IV, V, and VI) and these are further incorporated into reciprocal thalamic structures, both thalamic “core” nuclei, and the overlaying inhibitory thalamic layer termed nucleus reticularis thalamus (NRT). We study how these added structures further interact with the superficial layer L-FLIC structure to perform an enlarged set of algorithmic operations. In particular, we show that for each of the identified fundamental L-FLIC operations, the additional layers enable the system to perform iteratively and hierarchically, i.e., successive iterations of clustering or component analysis. This section discusses the competitive circuit activity, summarizing and adopting the core ideas of the framework introduced by Rodriguez et al. [30].

The core thalamic nucleus first relays static input to the middle cortical layer (layer IV), which then transmits the information apically to the superficial layer (II-III), where the L-FLIC operations occur. While local lateral inhibition is present within the L-FLIC assemblies in the superficial layers, synaptic change over multiple trials eventually causes excitatory neurons to generate nearly identical responses to similar inputs from the middle layers, as already described. This procedure corresponds to clustering.

The superficial layers then activate the deep layers (V–VI) in a topographic manner. Feedback from layer VI is then sent to both the thalamic reticular nucleus (NRT) and the core thalamus. The NRT inhibits portions of the core thalamic cells that correspond to just those cells already activated by the previous input. Due to the topographic organization, the inhibited portions also directly map back to the specific input regions that were just processed. This inhibition is long-lasting, persisting for hundreds of milliseconds [42,43,44]. As a result, whenever the same input is re-sensed, since it still is present in the environment, then only the uninhibited cells can be activated. That is, on successive sensory “samples” over time of a given input, first one set of cells is activated in thalamus (and then cortex), and then a quite different set of cells is activated on the second sampling, since the first set of target cells is now inhibited. We show that computationally, these sequential activities correspond to hierarchical clustering. We denote this process as lateral inhibition-hierarchical clustering (LI-HC).

LI-HC begins with an initial clustering and proceeds divisively through multiple learning cycles, each employing identical clustering procedures connected by “input masking” (the concepts underlying iterative masking are discussed in detail in Appendix A.1). In this paper, we refer to a “clustering module” as a complete clustering procedure that does not involve any input masking—that is, a plain, single-hierarchical clustering process. We represent neural synaptic activities using vectorized notation for simplicity. Given an external input set *X* of size *n*, where each data point is a *p*-dimensional column vector, we define a candidate winner set *W*, composed of a sufficiently large number of *p*-dimensional column vectors representing synaptic weight profiles. In this formulation, each excitatory neuron is represented by its vector of synaptic weights from the external input population, effectively characterizing its afferent connectivity. The similarity between an input vector and a neuron’s synaptic weight vector is evaluated using cosine similarity, which serves as the selection metric for competitive activation. Each input selects the weight vector with which it has the highest cosine similarity, designating it as the “winner”—that is, the neuron receiving the strongest effective synaptic drive from that input. Within each clustering module operating on a single input *x*, the selected weight vector *w* is typically unique; however, ties may arise when multiple candidates achieve the same maximum cosine similarity. Thus, the selection rule that enforces a single winner is termed winner-take-all. The selected “winner” is subsequently updated iteratively following a competitive learning rule:

w←w+r·(x−w)

where *r* is the learning rate, and x∈X. Or, equivalently, it can be written as $w_{i+1}=w_{i}+r\cdot \left(x-w_{i}\right)$ to indicate the *i*th update step. At each iteration, the winning weight vector is updated in the direction of the input vector *x*, thereby further increasing its alignment with the current input. Over iterations, the weight vectors gradually align with the input vector *x*. As this process is sequentially repeated for all data points, weight vectors associated with spatially similar inputs converge toward their mean, forming well-separated clusters. This batch convergence is established and justified in the Appendix A.2.

After initial clustering, due to the long-lasting inhibition from GABAergic NRT overlaying the core thalamic cells, the next sample of the same static input will contain only the secondary portion of input activity that was not shared among the members of the established cluster. Now the previous winners and their corresponding input portions become dormant. This means that the input is partially “masked” for further learning, and the previously learned feature is “forgotten” by presently active excitatory neurons within L-FLIC.

They are now only likely to be activated by the remaining unmasked information. This masking initiates a transition across cycles in the learning process. Following the same learning rule, the “active remainder” of the input actions will choose a different set of excitatory target neurons compared to whatever was selected in the previous cycle. After training, the new neural weight vector will point from the parent cluster’s (determined in the previous cycle) centroid toward the unique features present in this specific input instance. This process is known as sub-clustering, a step following the initial clustering in LI-HC. The training process is simplified in the Algorithm 1. This will be shown to be an elaboration and variant of a mechanism studied by Rodriguez et al. [30].
**Algorithm 1** LI-HC     **Input**: input data vectors *X*, initial weight vectors *W*     **Output**: resulting weight vectors *W* as centroids     **for** hierarchy 1,2,3… **do**               ▹ loop over hierarchies           **for** epoch 1,2,3… **do**              ▹ within each hierarchy                  **for** x∈X **do**                        $w=argmax(x^{T}W)$        ▹ here, *x* and *W* are normalized.                        w=w+(x−w)·r                  **end for**           **end for**          X=X−w                      ▹ input masking     **end for**

**Figure 2:**
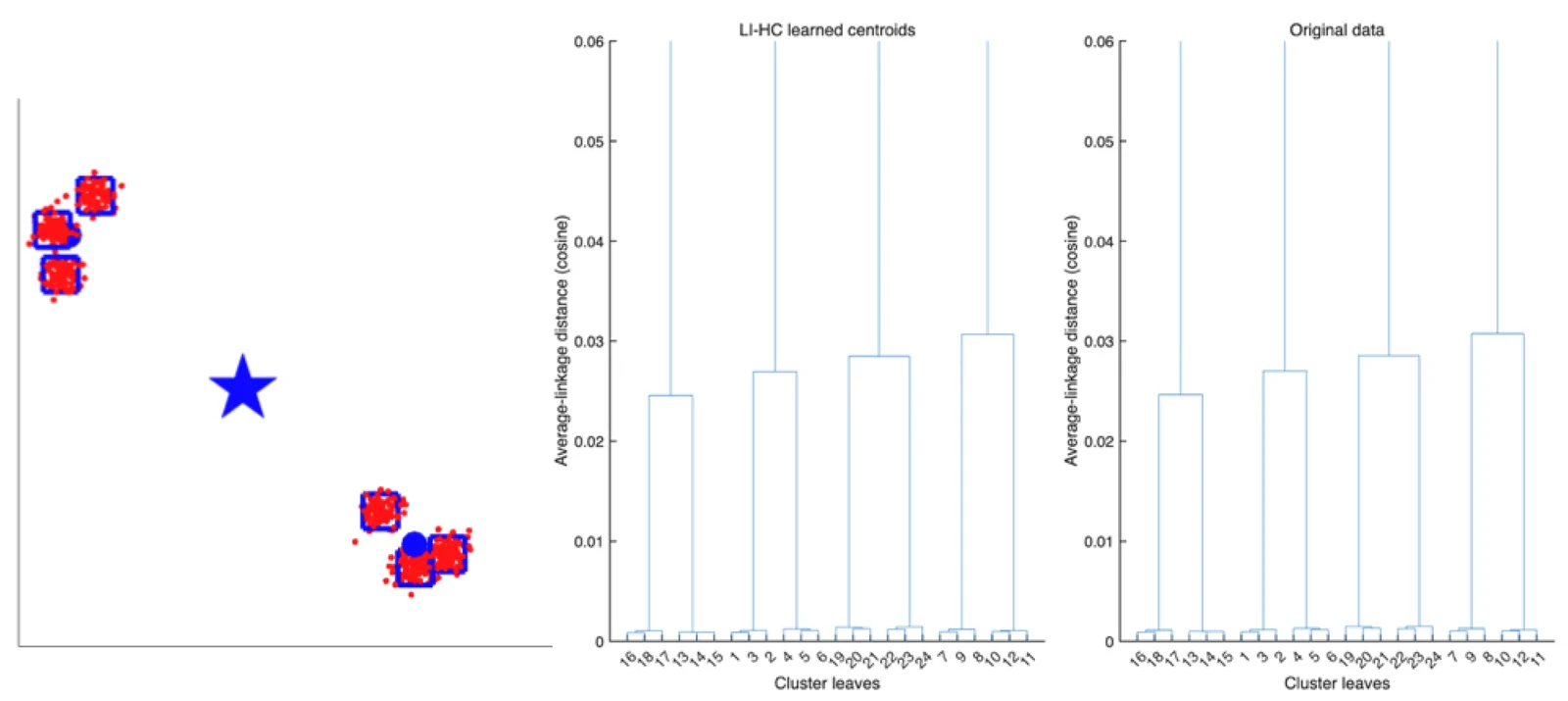
Demonstration of LI-HC. The leftmost figure presents the graphical outcome of LI-HC applied to a dataset containing two main clusters, each further subdivided into three sub-clusters. In this visualization, the star, dots (the upper left dot is partially overlayed under the other symbols), and hollow squares represent the resulting weight vectors after training with LI-HC, serving as centroids at different hierarchical levels. The middle and rightmost figures display dendrograms constructed from the learned centroids by LI-HC and from the original data, respectively. Notably, the hierarchical cut-offs observed along the y-axes exhibit a high degree of similarity, indicating that the learned centroids effectively capture the hierarchical categories of the original data.

Geometrically, “input masking” is essentially vector subtraction: the input subtracts the winners from all previous cycles they have won, with the head of the previous winning vector becoming the tail of the current winner. In other words, input masking is equivalent to origin shifting, which concludes the previous cycle of clustering as well as initiates the next round of hierarchical clustering. This learning process ends when there is no further separable data or upon a pre-defined termination criterion. Ultimately, all the selected and updated winners become the final centroids for each hierarchy of clusters in the process. Figure 2 shows a sample result of LI-HC over a synthetic input set, intentionally designed to exhibit clearly separable hierarchical cluster structure (the original result was in 3D; in Figure 2, we rotate the plots to improve the presentation of the centroids’ relative positions).

Furthermore, we compare LI-HC with the standard Lloyd *K*-means algorithm [45] on the same hierarchical synthetic dataset. Clustering quality is evaluated using three external metrics—Purity, Normalized Mutual Information (NMI), and Rand Index (RI)—and three internal metrics—Silhouette Coefficient (SC), Calinski–Harabasz Index (CHI), and Davies–Bouldin Index (DBI). The external metrics are computed against the ground-truth labels corresponding to each hierarchical level, while the internal metrics are computed from the resulting cluster geometry.

For the hierarchical *K*-means comparison, we use a divisive procedure matching the structure of the synthetic data: K=4 at the first level, followed by two-way subdivision of each parent cluster at the second level, and three-way subdivision at the third level, yielding 4, 8, and 24 clusters respectively. Table 1 reports the resulting metrics for both methods at all three hierarchical levels.

## 3. Cooperative Circuit Activity—AIME Algorithm

A small modification to the L-FLIC circuit—specifically, weakening the inhibition in the superficial layer until it becomes negligible—shifts local feedback dynamics from competitive to cooperative activity patterns. From a computational perspective, this transition transforms the circuit’s operation from LI-HC to a PCA-like feature extraction process. We formalize this behavior by introducing the AIME algorithm (Antipodal Iterative Mean Estimation).

AIME learns weight vectors (which mathematically represent neurons) through a two-stage procedure, each corresponding to a distinct form of neural feedback. The first stage, motivated by cooperative activity in L-FLIC, adjusts the weight vectors toward the dominant directions of variation in the input data. The second stage, inspired by additional layers of cortical processing, operates hierarchically to recover a complete set of dispersion directions.

### 3.1. Stage 1: Identification of Initial AIME Components

In stage 1 (the first learning cycle), AIME is applied directly to the original input data until convergence, with the primary objective of identifying directions of maximal data dispersion. During this process, each weight vector is updated independently toward directions induced by the data distribution. The inclusion of the sign() function ensures that the learned vectors maintain acute alignment with these directions, effectively resolving directional ambiguity while preserving orthogonality where appropriate.

At convergence, the algorithm produces a set of learned weight vectors that capture the dominant directional structure of the input. Importantly, it is not the specific magnitudes or orientations of these vectors that are of interest, but rather the axes (i.e., one-dimensional subspaces) on which they lie. We refer to these axes as the AIME components. Unlike classical formulations that restrict the number of components, stage 1 allows multiple AIME components to emerge. Throughout this paper, the terms “lines,” “axes,” and “AIME components” are used interchangeably to denote these underlying directional structures.

### 3.2. Stage 2: Identification of Remaining AIME Components

Unlike the single-cycle computation in stage 1, stage 2 consists of multiple stage-1-style learning cycles (hereafter referred to as “cycles”). In stage 2, AIME performs a sequence of hierarchical computations to extract additional components beyond those identified in stage 1. Rather than computing orthogonal directions in a single step, the algorithm iteratively transforms the input data and reapplies the stage 1 learning procedure.

Following each cycle, the algorithm extracts one or more directions that lie in the orthogonal complement of the subspace spanned by components from all preceding cycles. Consequently, orthogonality is enforced across loops, but not necessarily among components obtained within the same cycle. This process is analogous to the sequential extraction of principal components (PCs), where each subsequent component captures residual structure not explained by earlier ones.

For a *p*-dimensional input, stage 1 identifies the dominant component(s), while stage 2 performs up to p−1 additional cycles to extract the remaining components sequentially. Specifically, at the beginning of the *c*-th cycle (2≤c≤p), the algorithm applies an “input masking” step by projecting the data onto the subspace orthogonal to the components identified in previous cycles. As a result, the variance explained by earlier components is removed, and the transformed data represents the residual structure of the original input.

**Figure 3:**
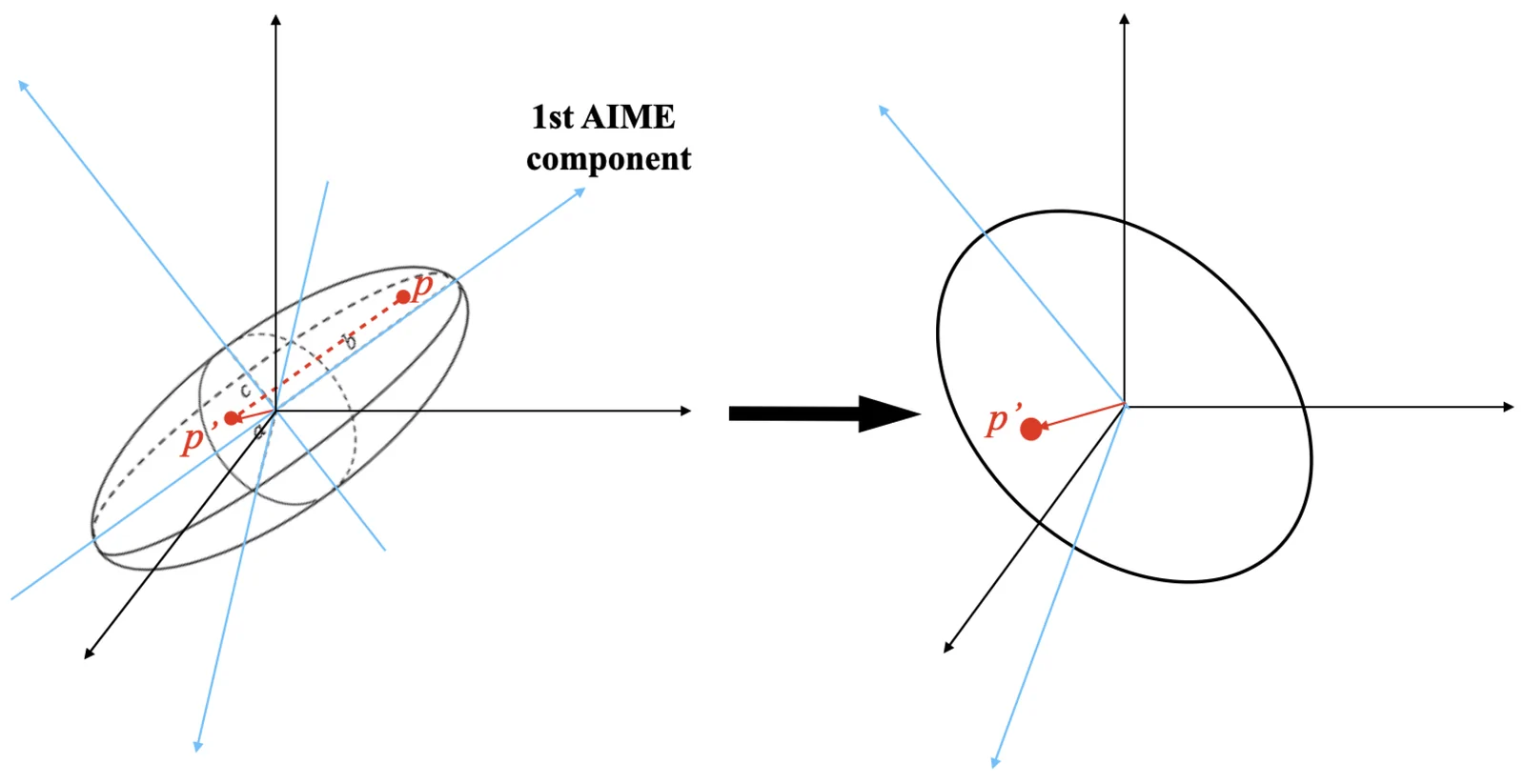
A geometric illustration of “input masking” using the first AIME component. The left subfigure shows data points distributed within an ellipsoid, where the polar radius *b* represents the first AIME component identified in the initial learning cycle. To compute the next AIME component, data points are projected onto a plane orthogonal to *b*, corresponding to the ellipsoid’s cross-section through the origin. For a data point *p* and the first AIME component *v*, the projection is calculated as $p^{\prime }=p-v\cdot \frac{p\cdot v}{{\left|v\right|}^{2}}$. The right subfigure demonstrates that this transformation reshapes the data into a 2D “circular region” within the 3D space, facilitating further analysis.

The stage 1 learning procedure is then applied to this masked data, enabling the algorithm to identify directions of maximum spread within the residual subspace. Repeating this process across cycles yields a hierarchical decomposition of the data and enables recovery of the full set of AIME components. Figure 3 illustrates how the data is progressively transformed to reveal deeper AIME components. The complete AIME algorithm with a single neural weight vector is provided in Algorithm 2.
**Algorithm 2** AIME with a single weight vector     **Input**: input data matrix *X*, initial weight vector *w*, learning rate *r*     **Output**: learned weight vector $w_{c}$ for each cycle *c*     **for** c∈1,2,3,4…p **do**                    ▹ *p*: total dimensions           reset *w*           **while** not converged **do**                 shuffle *X*                 **for** x∈X **do**                        $d=\mathrm{sign}(x^{T}w)$             ▹ sign( ) returns 1, −1, or 0                    w=w+(d·x−w)·r                 **end for**           **end while**           $w_{c}=w$           $X=X-v\cdot \frac{X^{T}w_{c}}{|w_{c}{|}^{2}}$     **end for**

## 4. Theoretical Analysis of AIME

Having introduced the AIME algorithm, we now present a theoretical analysis of its computational behavior. The goal of this section is to develop a mathematical framework that explains and supports the empirical behaviors observed in the simulation results. Rather than providing a complete axiomatic derivation, the analysis offers theoretical reasoning that helps interpret why the proposed algorithm exhibits the observed convergence properties under the proposed biological framework. A successful implementation of the algorithm relies on the following assumptions.

1. We assume that the input set is mean-centered and no data point is exactly located at the origin.
2. The dimensionality of the neural weight vectors and the size of the input dataset are assumed to be sufficiently large (this assumption is discussed further below).
3. The input data are assumed to contain meaningful structure, i.e., they are not perfectly uniform across all directions of the input space. Equivalently, the data variances are not identically distributed among all principal components. Otherwise, the resulting behavior becomes degenerate.

### 4.1. Definitions and Preliminary Formulation

Consider the input set $X_{n\times p}$ (for simplicity, we write it as *X*), which contains *n* data points $\left\{x_{1},x_{2},\ldots ,x_{n}\right\}$, each with *p* dimensions. The Antipodal Augmentation of *X* is the union of *X* and its negation, −X, and is written as

$$\mathrm{Aug}\left(X\right)=\left\{x_{1},\;x_{2},\;\ldots ,\;x_{n},\;-x_{1},\;-x_{2},\;\ldots ,\;-x_{n}\right\}.$$

**Figure 4:**
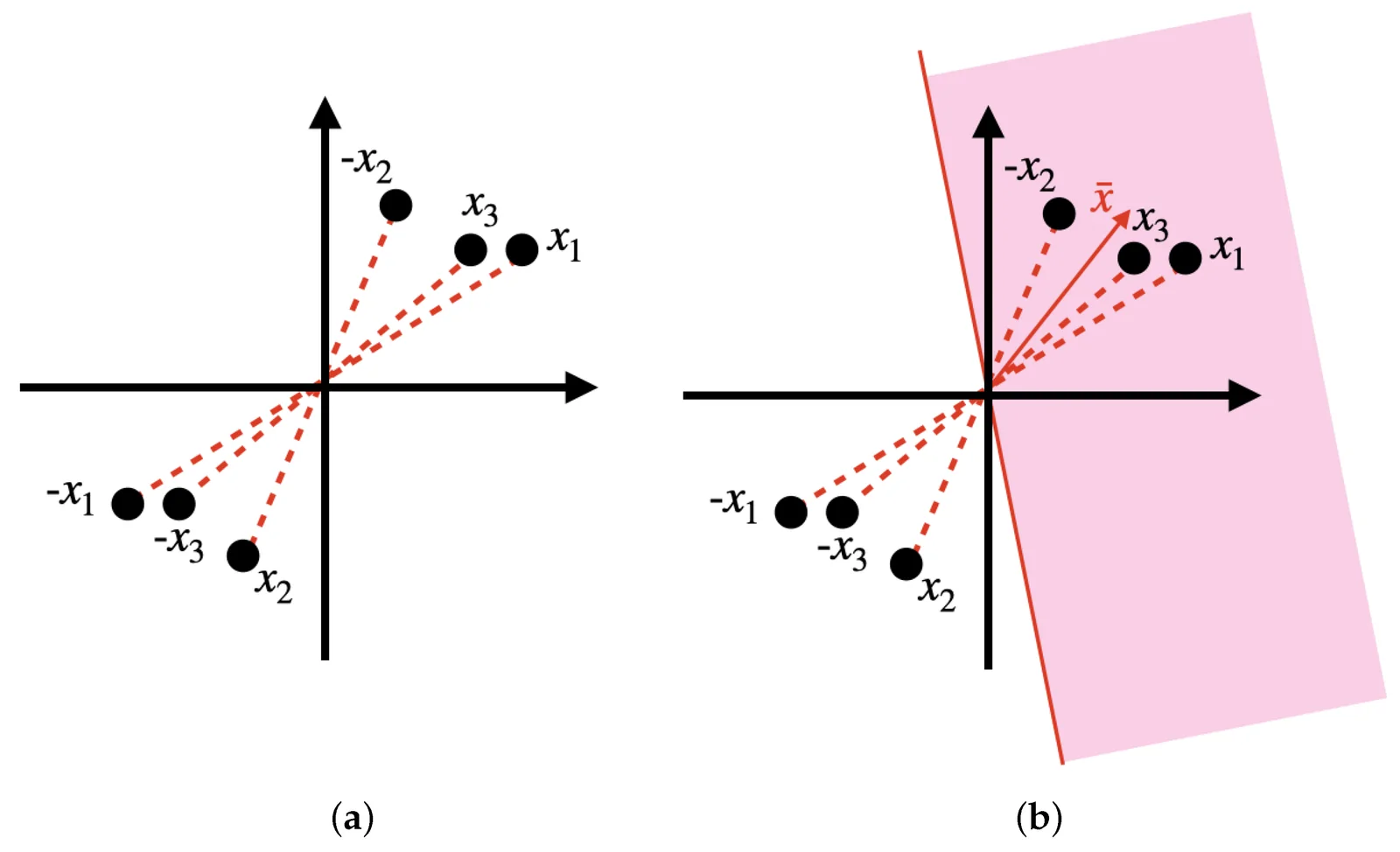
Antipodal augmentation of a sample input set $X_{3\times 2}$ and an acute partition. (**a**) shows the antipodal augmentation of *X*, where X= {$x_{1},x_{2},x_{3}$} and Aug(X)= {$x_{1},x_{2},x_{3},-x_{1},-x_{2},-x_{3}$}. In (**b**), the pink panel is an acute partition of Aug(X) with the corresponding $\tilde{X}=$ {$x_{1},-x_{2},x_{3}$}. Their mean vector, $\bar{x}=\left(x_{1}-x_{2}+x_{3}\right)/3$, is acute to each of these vectors.

Geometrically, $\mathrm{Aug}\left(X\right)\in R^{p}$ represents the bi-directional expansion of *X* in the coordinate, where each point is mirrored across the origin (see Figure 4a). In an *p*-dimensional Euclidean space $R^{p}$, a hyperplane *H* passing through the origin is defined as:

$$H=\{x\in R^{p}|w\cdot x=0\}$$

where w≠0 is a normal vector. This hyperplane bipartitions $R^{p}$ into two open half-spaces:

$$H^{+}=\left\{x\in R^{p}|w\cdot x>0\right\},\;H^{-}=\left\{x\in R^{p}|w\cdot x<0\right\}$$

In Aug(X), a choice of one vector from each antipodal pair defines a candidate partition. We denote the selected vectors by $\tilde{X}={\left\{{\tilde{x}}_{i}\right\}}_{i=1}^{n}$, where each ${\tilde{x}}_{i}$ is either $x_{i}$ or $-x_{i}$. Two complementary selections related by a global sign reversal represent opposite orientations of the same underlying directional structure. In the AIME update rule, the sign(·) function determines the orientation selected from each antipodal pair relative to the current weight vector.

We define an iteration as one complete training epoch over the input dataset. Across iterations, changes in the weight vector may alter the selected set $\tilde{X}$. From this perspective, AIME can be interpreted as an iterative procedure that updates both the weight vector and its associated antipodal selection until the selection becomes stable under the specified convergence criterion.

A partition of Aug(X) is called an acute partition (denoted as ap) if its associated vectors ${\tilde{x}}_{i}˜$ form acute angles with their mean (see Figure 4b). These vectors are referred to as the acute-partition vectors, and the mean of these vectors is called the acute-partition mean. For simplicity, we say that the acute-partition vectors are “acute to” their acute-partition mean. In Aug(X), each vector $x_{i}$ has an associated antipodal pair, consisting of $x_{i}$ and $-x_{i}$. These two vectors lie on the same straight line and the collection of all such lines, formed by antipodal pairs, intersect at the origin. Each of these lines is referred to as the line of $x_{i}$ or equivalently, the line of $-x_{i}$. To operationalize this definition, Algorithm 3 provides a constructive heuristic for identifying an acute partition of an input set satisfying the assumptions above. The procedure repeatedly reverses vectors that are not acute to the current mean and recomputes the mean until the selected partition stabilizes. This construction provides the analytical basis for the convergence reasoning developed in the following subsections. Additional mathematical reasoning concerning the existence of acute partitions is provided in Appendix A.3.
**Algorithm 3** Acute Partition Finding Heuristic     **Input**: Input set *X*     **Output**: Acute partition of Aug(X), containing $\tilde{X}$.     Start with a random Aug(X), with vector set $\tilde{X}$     **for** c=1,2,3,… **do**                                            ▹ Iterative step           Compute the mean vector $m=\mathrm{mean}(\tilde{X})$           Identify subset $X_{1}\subseteq \tilde{X}$ such that for each $x_{i}\in X_{1}$, the dot product $x_{i}^{T}\cdot m\leq 0$                       ▹ Find vectors forming non-acute angles with *m*           Update $\tilde{X}=\left(\tilde{X}\setminus X_{1}\right)\cup \left(-X_{1}\right)$                             ▹ Flip the directions of vectors in $X_{1}$     **end for**

### 4.2. Single-Neuron Convergence

We first consider the behavior of a single neuron, represented by one neural weight vector. Under the AIME update rule, the orientation of each input vector is determined relative to the current weight vector, resulting at each iteration in a selected set $\tilde{X}$ containing one vector from each antipodal pair in Aug(X). The weight vector is then updated toward the mean of this selected set. As the iterations proceed, the weight vector and the corresponding selection $\tilde{X}$ are updated jointly until the selected vectors become stable. At such a stable configuration, the weight vector approaches the mean of an acute partition, for which the mean forms an acute angle with each vector in the associated $\tilde{X}$. We summarize this basic convergence behavior as follows.

**Proposition** **1** (single-neuron convergence to an acute-partition mean). *Under the AIME algorithm, a single randomly initialized weight vector iteratively approaches the mean of its associated selected vectors. When the antipodal selection $\tilde{X}$ becomes stable, the resulting weight vector approaches the mean of an acute partition of Aug(X). Detailed reasoning can be found in Appendix A.4.*

This characterization identifies a stable form of single-neuron convergence, but does not imply that the corresponding acute partition is unique. Depending on the spatial organization of the input data, multiple acute partitions and their associated means may exist. The remaining question is therefore not whether a stable acute-partition mean can be reached, but which of the possible acute-partition means is preferentially approached by the AIME dynamics.

Our simulations, together with the update behavior of the algorithm, suggest that convergence preferentially occurs toward acute partitions whose selected vectors are more strongly concentrated around their mean. Intuitively, such a configuration provides greater directional consistency among the selected vectors, making its mean more stable under successive AIME updates. We refer to an acute partition exhibiting the strongest such concentration as the strongest acute partition, and its mean as the strongest acute-partition mean (SAPM). The SAPM is therefore introduced as a computational characterization of the empirically observed convergence tendency, rather than as an independently derived closed-form solution.

Based on this empirical observation and the corresponding algorithmic reasoning, we summarize the preferential convergence behavior of a single neural weight vector as follows.

**Proposition** **2** (preferential convergence toward the SAPM). *For an input set with a distinguishable strongest acute partition, repeated AIME updates tend to drive a randomly initialized single neural weight vector toward the corresponding SAPM. Detailed reasoning can be found in Appendix A.5.*

### 4.3. Multi-Neuron Convergence

We now extend the analysis from a single neuron to a population of independently initialized neural weight vectors. Each weight vector follows the single-neuron convergence behavior described in the previous subsection, evolving toward the mean of a stable acute partition under the AIME update rule. Consequently, the overall population behavior is determined not by interactions among neurons, but by the geometric organization of the input data, which determines the distribution of stable acute partitions available for convergence.

From a computational perspective, the strongest acute-partition mean (SAPM) and the leading principal component are motivated by closely related objectives. Both seek to identify dominant directions that best capture the global organization of the input data, although they quantify this objective differently. The SAPM is defined through the directional concentration and stability of acute partitions under the AIME dynamics, whereas PCA identifies directions that maximize the variance of the projected data. Thus, while the two formulations are fundamentally different, they are both intended to characterize the principal directional structure of the dataset.

Guided by this observation, we next consider the behavior of a population of independently trained neural weight vectors. If the input data possess a clearly distinguishable strongest acute partition, most weight vectors tend to converge toward the corresponding SAPM, producing a single dominant AIME component. When several acute partitions exhibit comparable directional concentration, different weight vectors may instead converge toward different SAPMs, giving rise to multiple AIME components. The resulting population distribution therefore reflects the underlying directional organization of the data rather than interactions among the neurons themselves.

Interestingly, our simulation results consistently show that the recovered AIME components exhibit remarkably strong alignment with the principal components of the same dataset. When a unique dominant direction exists, the dominant AIME component is closely aligned with the leading principal component. When multiple comparable directions exist, multiple AIME components emerge, among which a subset exhibits strong correspondence with the principal-component directions. These observations motivate the computational connection between AIME and PCA explored throughout this work.

Importantly, this relationship is established through the observed computational behavior of the algorithm together with the theoretical reasoning developed in the previous subsection, rather than through a complete algebraic derivation. We therefore interpret the correspondence between AIME and PCA as a convergence of computational behavior: two fundamentally different computational formulations that nevertheless recover highly consistent representations of the dominant directional structure of the input data.

From an algorithmic perspective, employing a population of independently initialized neural weight vectors enables AIME to reveal both dominant and competing directional structures present in the data. This behavior is also consistent with the biological motivation of the cooperative circuit, in which weakened feedback inhibition allows multiple excitatory neurons to remain simultaneously active and represent different aspects of the input distribution.

### 4.4. Summary of the Theoretical Framework

The theoretical framework developed in this section provides a computational interpretation of the AIME algorithm rather than a complete axiomatic derivation. The key principles of AIME are reflected in its name:

- **A**ntipodal: learning is performed on the antipodally augmented input set, Aug(X).
- **I**terative: neural weight vectors are updated iteratively until stable antipodal selections are reached.
- **M**ean: each weight vector converges toward the mean of a stable acute partition.
- **E**stimation: a population of independently trained weight vectors recovers one or more stable AIME components that characterize the dominant directional structure of the data.

The theoretical reasoning presented in this section, together with the simulation results in the following sections, suggests a close computational relationship between AIME and principal component analysis. Although the two methods arise from fundamentally different formulations, they both recover highly consistent representations of the dominant directional organization of the input data. Accordingly, the relationship between AIME and PCA should be interpreted as a convergence of computational behavior rather than as a strict algebraic equivalence.

Finally, we emphasize that AIME is not derived from principal component analysis or other classical statistical methods. Rather, it originates from the biologically motivated cooperative dynamics of the proposed cortical circuit. The correspondence with PCA therefore emerges as an empirical and computational observation supported by theoretical reasoning, rather than being imposed by construction.

## 5. Simulation Methods

### 5.1. Data Preparation

Input sets: To evaluate the behavior of AIME and its relationship to principal component analysis (PCA), we conduct simulations on two datasets with contrasting variance structures: the Iris dataset and a synthetic dataset.

The Iris dataset [46] is a well-known benchmark consisting of 150 samples from three species (Iris setosa, Iris versicolor, and Iris virginica), each described by four numerical features: sepal length, sepal width, petal length, and petal width. In this work, all four features are treated as continuous variables. Table 2 reports the corresponding explained variances. Notably, the Iris dataset exhibits a pronounced decay in variance across principal components.

In contrast, we consider a synthetic dataset with 300 samples and 50 numerical features [47], designed to exhibit a more uniform variance distribution. As shown in Table 2, the explained variances decrease more gradually across components, indicating a less dominant leading direction.

These two datasets provide complementary settings: one with a strongly dominant principal direction and one with a more evenly distributed variance structure, allowing us to examine how AIME behaves under different dispersion regimes. These datasets constitute the primary simulation experiments analyzed in detail in the following sections. In addition, to provide a complementary demonstration of scalability to higher-dimensional real-world data, we apply AIME to the Modified National Institute of Standards and Technology (MNIST) image dataset [48]. This supplementary experiment is presented in the Conclusions and Discussion section and is intended as a scalability demonstration rather than as a third primary benchmark.

To further evaluate the generalizability of AIME beyond the two primary simulation datasets, we additionally consider two real-world benchmark datasets from the *UCI Machine Learning Repository*: the Wine dataset [49], containing 178 samples and 13 numerical features, and the *Wisconsin Diagnostic Breast Cancer (WDBC)* dataset [50], containing 569 samples and 30 numerical features. Because the native feature scales differ substantially in both datasets, the numerical features are standardized to zero mean and unit variance before analysis. For these additional benchmarks, we focus on recovery of the first three principal-component directions and compare AIME with the classical Oja learning rule [16] and Sanger’s Generalized Hebbian Algorithm (GHA) rule [51], biologically inspired neural algorithms for principal-component extraction. These experiments are intended as complementary validation rather than full replications of the detailed Iris and synthetic-data analyses.

### 5.2. AIME Component Determination

After each learning cycle, the obtained AIME components relate to the leading principal component (PC) in two distinct ways: (1) When the input data exhibits a dominant direction of variance, the learned weight vectors tend to align along a single line. In this case, the AIME component is uniquely determined, and the leading PC is collinear with this line. (2) When the input data lacks a clearly dominant direction and is more evenly distributed, the learned weight vectors may instead align along multiple distinct lines (i.e., multiple AIME components are obtained within a single learning cycle). These lines correspond to directions with comparable explained variances, and the leading PC is approximately aligned with one of them.

These two cases reflect whether the data is dominated by a single principal direction or exhibits a more distributed variance structure.

Given the potential complexity within each learning cycle, combined with the hierarchical structure of stage 2, determining the full set of AIME components involves two key steps:

- Step 1: Line identification within each cycle. The direct outputs of each learning cycle are the learned weight vectors, which typically form clusters in the weight space. Due to the antipodal symmetry of the learning dynamics, these clusters are expected to appear in pairs (with opposite signs) centered at the origin. Each such pair defines a line (i.e., an AIME component) passing through the origin. To identify these lines, clustering methods such as K-Means (or other user-specified approaches) are applied to group the learned weight vectors. The number of clusters *K* is selected based on the observed structure of the data. This step allows the extraction of aggregated directional structures and clarifies the organization of AIME components within each cycle.
- Step 2: Input masking with hierarchical inheritance.
  For each identified AIME component, the input data is projected onto the orthogonal complement of the corresponding direction, effectively removing the variance explained by that component. When multiple AIME components are obtained within a single cycle, this masking procedure is applied separately to each component, resulting in multiple transformed datasets.
  Subsequent learning cycles are performed independently on each transformed dataset. The resulting components are therefore conditionally defined with respect to their parent component, forming a hierarchical structure. As a result, components derived from different parent directions are not directly comparable, as they correspond to distinct residual subspaces. For example, given parent components $line_{A}$ and $line_{B}$, the components obtained from data masked along $line_{A}$ are not directly comparable to those derived from $line_{B}$.

### 5.3. Implementation Details and Reproducibility

To improve reproducibility, we summarize here the implementation settings used in the AIME simulations. The same underlying AIME update rule was used for all datasets, while several numerical parameters were selected conservatively according to the scale and dimensionality of each input dataset. These settings were used to obtain stable computational behavior rather than being optimized through a formal hyperparameter search.

Learning rate and training duration. A fixed learning rate was used within each dataset. For both the Iris and synthetic datasets, the learning rate was set to $r=10^{-4}$, whereas the substantially higher-dimensional MNIST experiment used a smaller learning rate of $r=10^{-7}$. These values were selected empirically to provide sufficiently small and stable weight updates; no optimization of the learning rate with respect to the reported PCA-correspondence metrics was performed. See Table 3 for details.

Training was performed using a fixed number of epochs rather than an error-tolerance stopping rule. Iris and synthetic-data experiments were trained for 400 epochs per learning cycle, while the MNIST experiment was trained for 500 epochs. These epoch counts were chosen conservatively to allow the learned weight directions to reach a stable regime. The convergence behavior over training is further illustrated in Figures 8 and 9, showing that the fixed training budgets extend beyond the main period of directional change.

Weight-vector population and initialization. A single neural weight vector is sufficient to execute the basic AIME update rule, as described in Algorithm 2. In the simulations, however, multiple independently initialized weight vectors were used to sample the population-level distribution of possible convergent directions and thereby reveal the organization of the resulting AIME components. We used 600 weight vectors for the Iris and synthetic-data experiments and 70 weight vectors for the MNIST experiment.

Excitatory neurons in the L-FLIC circuit exhibit sparse synaptic connectivity. To model this sparsity, we initialize weight vectors using a sampling scheme inspired by the hypergeometric distribution, as suggested by Felch and Granger [7]. Specifically, each weight vector is constructed by selecting a subset of active (nonzero) entries without replacement, resulting in a fixed level of sparsity across neurons. While the AIME algorithm is not restricted to this initialization and can operate with general random weight vectors, incorporating this structured sparsity improves biological plausibility and aligns the model more closely with known properties of synaptic organization.

Post-hoc identification of AIME components. The direct output of AIME is a population of learned weight vectors rather than a predefined number of components. As described in Section 5.2, the converged weight vectors frequently organize into visibly distinguishable directional groups. K-Means is therefore used only as a post-hoc summarization procedure to identify the centroids of these groups; it is not part of the AIME learning rule itself.

The value of *K* was selected empirically from the observed grouping structure of the learned weight vectors after three-dimensional projection, rather than through an elbow, silhouette, or other automatic model-selection criterion. Because each AIME component is bidirectional, one component generally corresponds to a pair of approximately antipodal clusters. Accordingly, the Iris simulations used K=2 for each identified component. For the synthetic data, K=2 was used when a single component was observed within a cycle and K=4 when two distinct components were observed. The MNIST demonstration used K=4, corresponding to two dominant bidirectional groups. This clustering step serves only to summarize the converged weight-vector population and does not alter the underlying AIME training dynamics.

Multi-seed reproducibility analysis. To assess the robustness of AIME with respect to random initialization, we additionally repeated the Iris and synthetic-data experiments across 100 independent random seeds. For each seed, the complete AIME procedure was rerun using the same learning rate, number of weight vectors, training duration, and post-hoc component-identification procedure described above.

For the Iris dataset, one dominant AIME component was identified in each learning cycle, and we recorded the absolute cosine similarity between that component and the corresponding principal component. For the synthetic dataset, multiple AIME components may emerge within a learning cycle; in this case, for each seed and cycle, we recorded the component exhibiting the strongest absolute alignment with the corresponding principal component. This cycle-level metric therefore evaluates whether AIME consistently recovers a PC-aligned component across independent initializations without requiring one-to-one correspondence among additional emergent branches.

For each dataset and learning cycle, the resulting 100 similarity values were summarized using the mean, standard deviation, and 95% confidence interval. These analyses are used specifically to evaluate initialization robustness and are reported separately from the single-seed simulations used for the detailed graphical analyses.

Learning-rate sensitivity analysis. To evaluate whether the reported results depend critically on the selected learning rate, we additionally performed a sensitivity analysis on the first learning cycle of the Iris and synthetic datasets, where a single dominant AIME component is recovered without subsequent component branching. The learning rate was varied logarithmically from $10^{-6}$ to 1, while the remaining implementation settings were held fixed. For each learning rate, we measured the absolute cosine similarity between the recovered AIME component and PC1.

Role of the neural weight-vector population. The number of neural weight vectors *q* is not treated as an optimization hyperparameter in AIME. Each neural weight vector executes the AIME update independently and in parallel; therefore, increasing *q* does not change the underlying learning dynamics or the convergence criterion of an individual neuron. In principle, even a single weight vector is sufficient to execute the AIME learning rule.

A larger *q* instead provides denser sampling of the possible convergent directions under different random initializations. Consequently, multiple learned weight vectors may converge to overlapping or nearby directions within the same AIME component. The component direction is subsequently represented by the centroid of these converged weight vectors, thereby averaging initialization-dependent variation within the corresponding directional group. Increasing *q* can therefore provide a more stable population-level estimate of the emergent components and improve the representation of possible branching structures, rather than increasing the correctness of the underlying single-neuron AIME computation. For this reason, we use a conservatively large population in the reported simulations rather than defining an “optimal” value of *q*.

### 5.4. Measurements

The validation of AIME is conducted using two complementary measurements, capturing both geometric and numerical properties of the learned components.

First, correlation is used to evaluate the geometric alignment between the true principal components (PCs) and the AIME components. Specifically, we compute the correlation between their corresponding direction vectors (namely, the centroids of the grouped learned weight vectors discussed in step 1) and consider only the absolute values, thereby measuring alignment irrespective of orientation (i.e., treating antipodal directions as equivalent). The resulting values range from 0 to 1, where values close to 1 indicate strong alignment. When multiple AIME components are identified, correlations are reported for each component.

Second, the cumulative explained variance (in %) provides a numerical assessment of how well the AIME components capture the dispersion of the data. This is computed by projecting the data onto the corresponding components, analogous to PCA. For high-dimensional datasets, the evaluation focuses on the selected leading components rather than exhaustively reporting all dimensions. For comparison with the corresponding AIME components, the explained variances of these selected PCs are normalized by their total variance, such that the selected subset sums to 100%. Thus, the reported values represent the relative distribution of explained variance within the selected component subset, rather than the percentage of total variance explained by the full-dimensional dataset.

## 6. Simulation Results

Summary: The results from the Iris dataset and the synthetic dataset reveal distinct patterns, clearly demonstrating the following:

1. Validity of AIME—AIME is an effective algorithm for estimating true PCs as well as extracting other meaningful components if needed.
2. Well-structured Iris Data—The Iris dataset has a clear structure, with contrasting concentrations along different dimensions. As a result, AIME produces lines closely matching with true PCs.
3. Noisy Synthetic Data—The synthetic dataset represents a noisier scenario, with data more evenly distributed and lacking clear spatial patterns. Consequently, AIME generates a family of components, one of which aligns with the true PCs.

**Figure 5:**
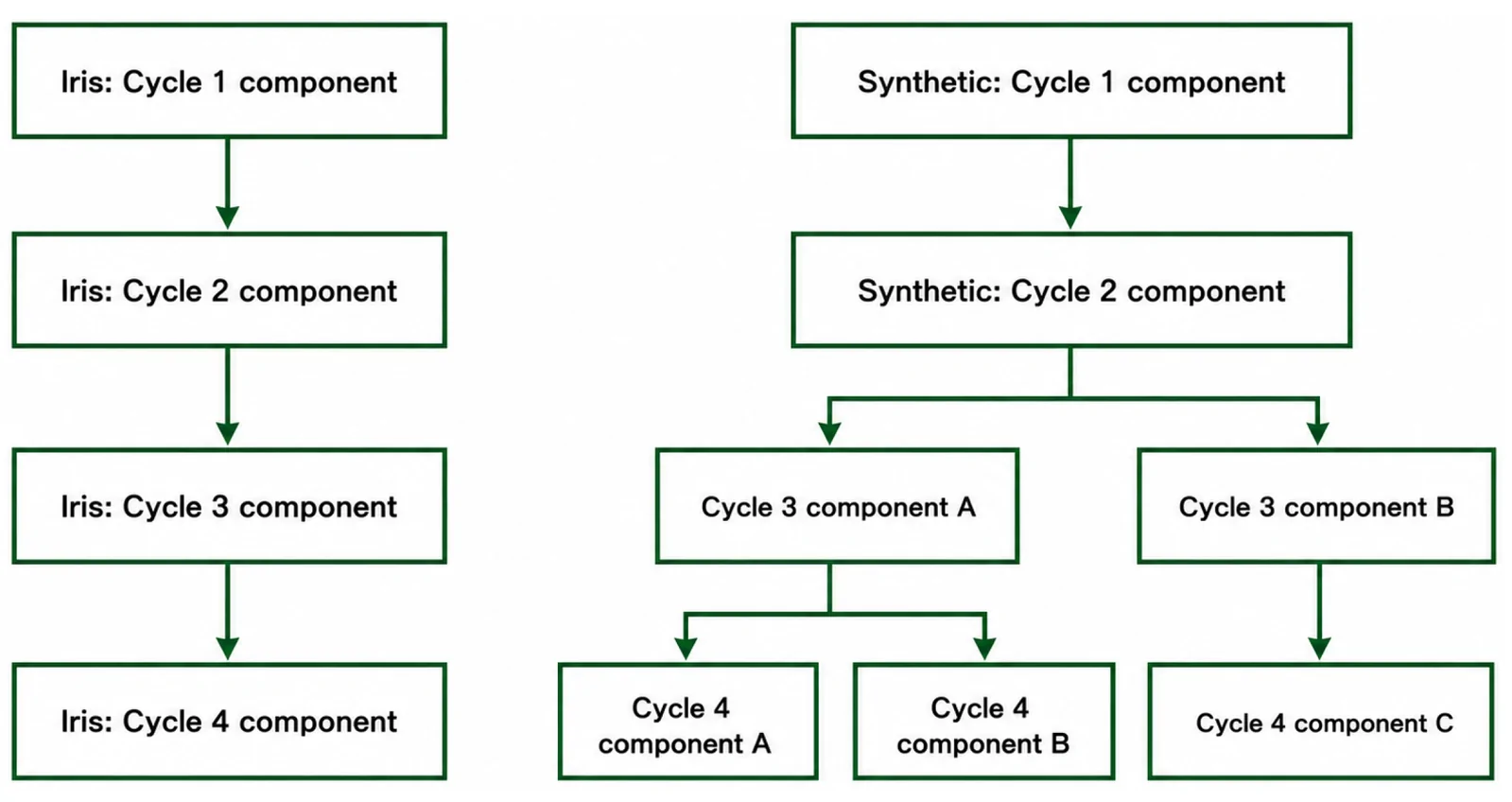
Learning progression of AIME across successive cycles for the Iris and synthetic datasets. The Iris dataset (**left**) yields a single recovered AIME component in each cycle, resulting in a linear learning trajectory. In contrast, the synthetic dataset (**right**) exhibits a branching structure: a single component is recovered in cycles 1 and 2, two components emerge in cycle 3, and three descendant components are obtained in cycle 4 through component-specific input masking. The branching structure illustrates that multiple AIME components can emerge from the residual data as successive dominant directions are removed.

Diagram: Figure 5 outlines the resulting lines (i.e., AIME components) produced by Iris and the synthetic data. Iris data’s dispersion is tight and the learned weight vectors roughly form a single AIME component after each cycle. With the synthetic data, which is more uniformly distributed across different axes, we obtain multiple AIME components. The diagram highlights input masking with inheritance.

**Figure 6:**
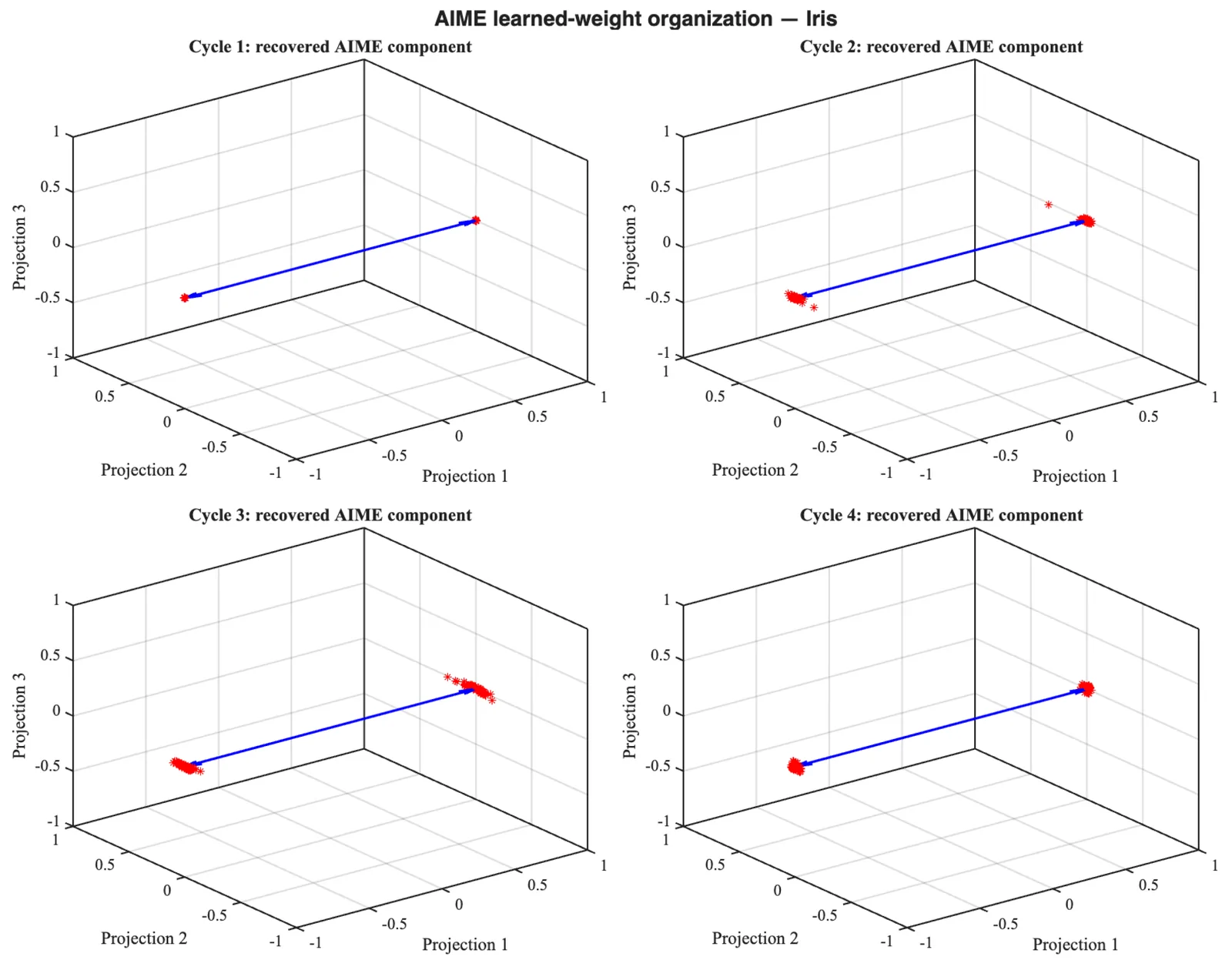
Visualization of the learned neural weight-vector organization across four successive AIME learning cycles on the Iris dataset. Each red star represents one independently initialized neural weight vector after convergence in the corresponding learning cycle, projected into three-dimensional space for visualization. Blue lines connect approximately antipodal centroid pairs identified by post-hoc K-means clustering (K=2), and therefore represent the recovered bidirectional AIME component in each cycle. Across all four cycles, the learned weight vectors organize predominantly around a single component direction.

**Figure 7:**
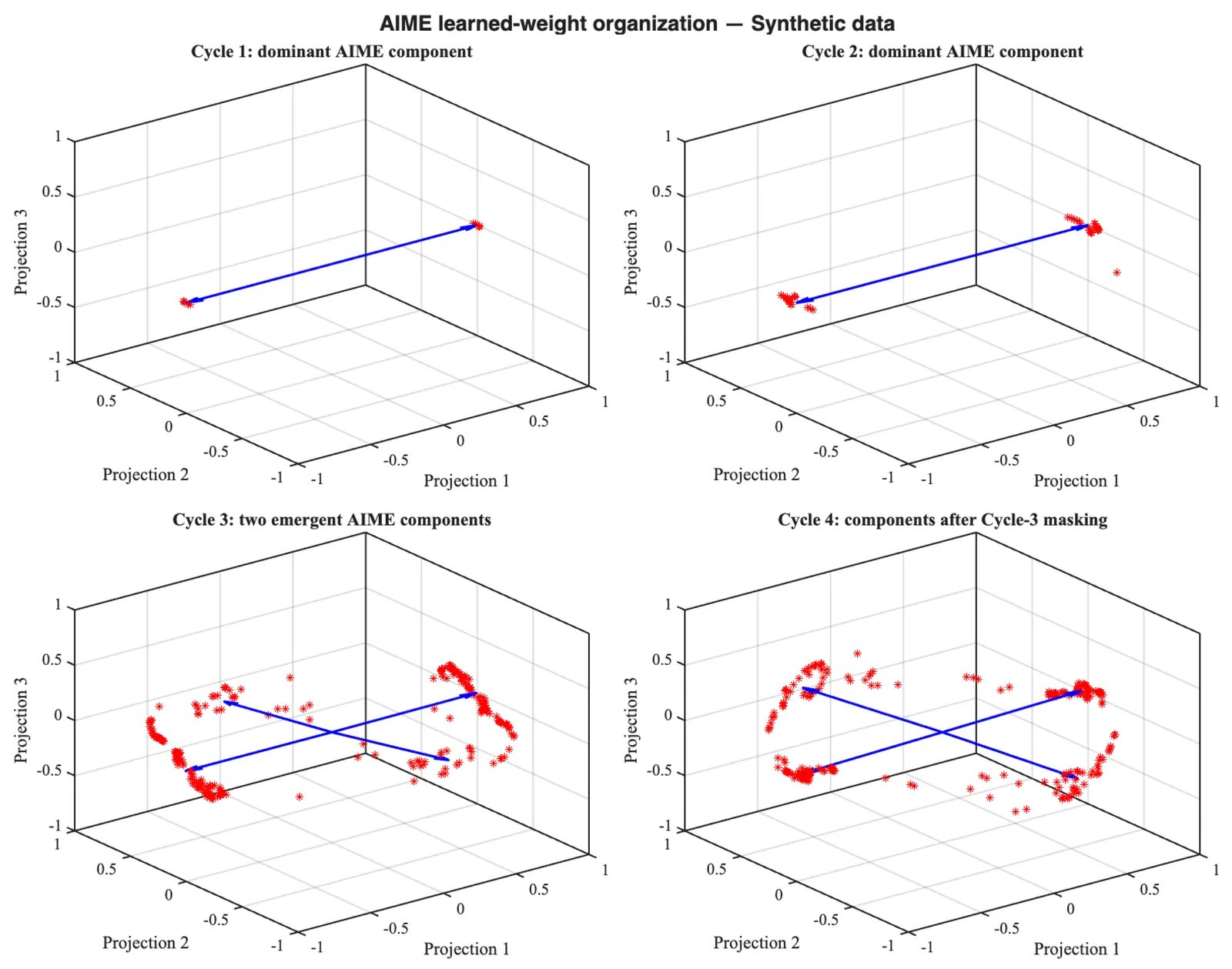
Visualization of the learned neural weight-vector organization across successive AIME learning cycles on the synthetic dataset. Each red star represents one independently initialized neural weight vector after convergence in the corresponding learning cycle, projected into three-dimensional space for visualization. Blue lines connect approximately antipodal centroid pairs identified by post-hoc K-means clustering and represent the recovered bidirectional AIME components. Cycles 1 and 2 exhibit a single dominant component, whereas multiple directional groups emerge in later cycles, illustrating the branching structure observed as successive dominant directions are removed from the input. Cycle 4 shows the result obtained after masking the selected Cycle-3 component used for the subsequent learning cycle.

Graphical outcomes: Figure 6 and Figure 7 visualize the internal organization of the learned neural weight vectors during AIME training. Unlike Figure 5, which provides a schematic illustration of the resulting AIME components, these figures present the actual learned neural weight vectors from which the AIME components are identified.

After each training cycle, AIME updates a population of neural weight vectors, which gradually self-organize into distinct groups. Each red star represents one independently initialized neural weight vector after convergence in the corresponding learning cycle. For visualization, these high-dimensional weight vectors are projected into three-dimensional space. The projected vectors naturally form visually identifiable clusters. To objectively identify the dominant directional structures, we apply K-means clustering to the converged weight vectors. The resulting cluster centroids occur as approximately symmetric pairs about the origin. Each centroid pair therefore defines a line passing through the origin, which is interpreted as one AIME component.

The number of recovered lines is consistent with the number of meaningful components suggested by the conceptual illustration in Figure 5. Figure 6 and Figure 7 therefore provide an empirical visualization of how the learned neural weight vectors organize into symmetric directional groups, from which the final AIME components are identified.

To further examine the convergence behavior of AIME, we track the directional evolution of representative neural weight vectors across learning epochs. Because the AIME components are identified post hoc from the converged weight-vector population using K-means clustering, the component centroids themselves cannot be directly tracked during training. Instead, after the final AIME components are identified, we select a representative neural weight vector associated with each final component and retrospectively trace its trajectory across epochs. Convergence is quantified by the absolute cosine similarity between the representative weight vector and the corresponding principal component of the input data. A value approaching 1 therefore indicates increasing directional alignment with that principal component. For cycles in which multiple AIME components emerge, the representative trajectories of the corresponding components are shown separately.

**Figure 8:**
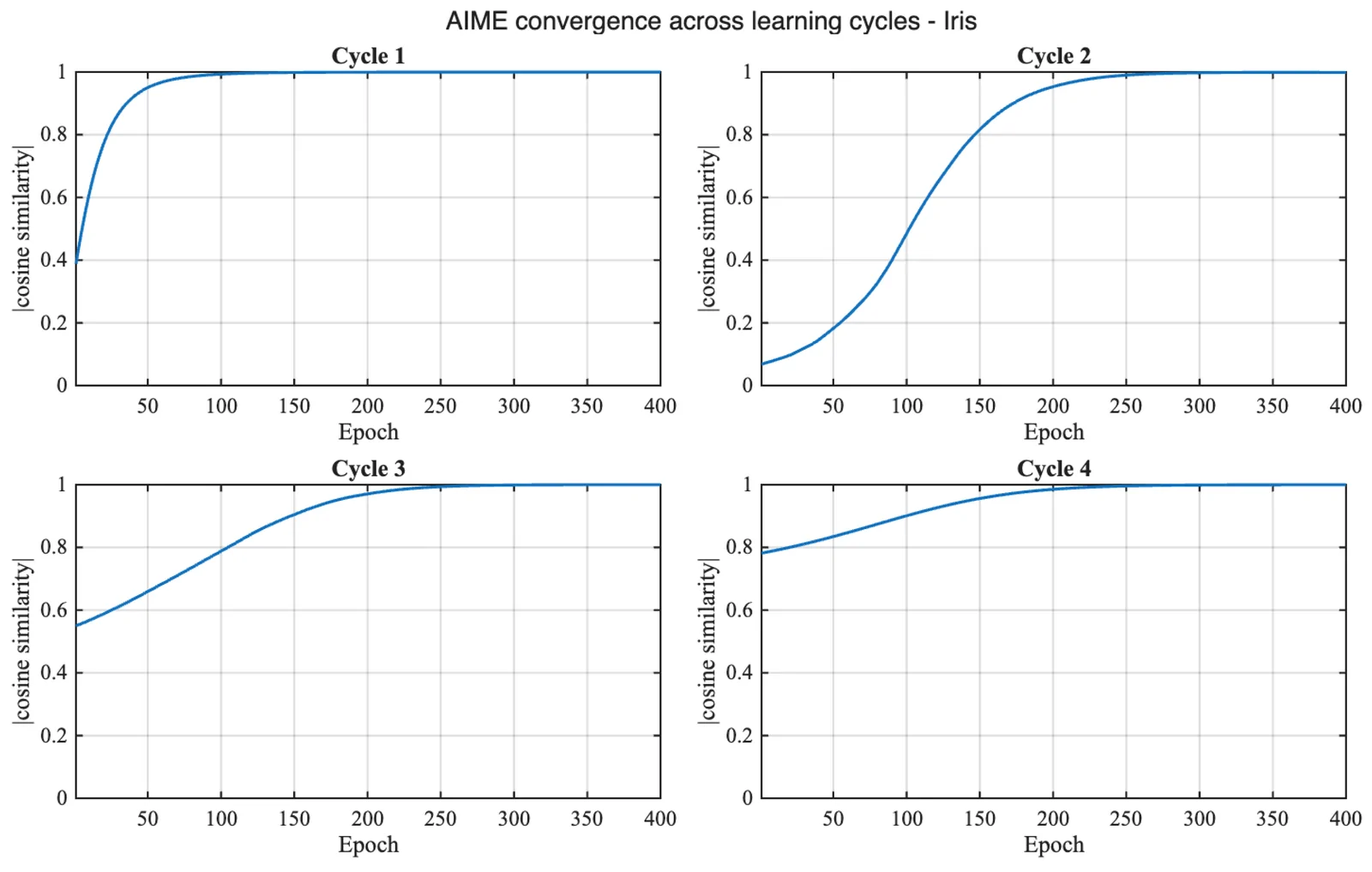
Convergence of AIME learning across four successive cycles on the Iris dataset. Each panel shows the absolute cosine similarity between the representative neural weight vector associated with the final AIME component and the corresponding principal component as a function of learning epoch. Across all four cycles, the similarity progressively approaches 1, indicating stable directional alignment between the learned AIME components and the corresponding principal directions.

**Figure 9:**
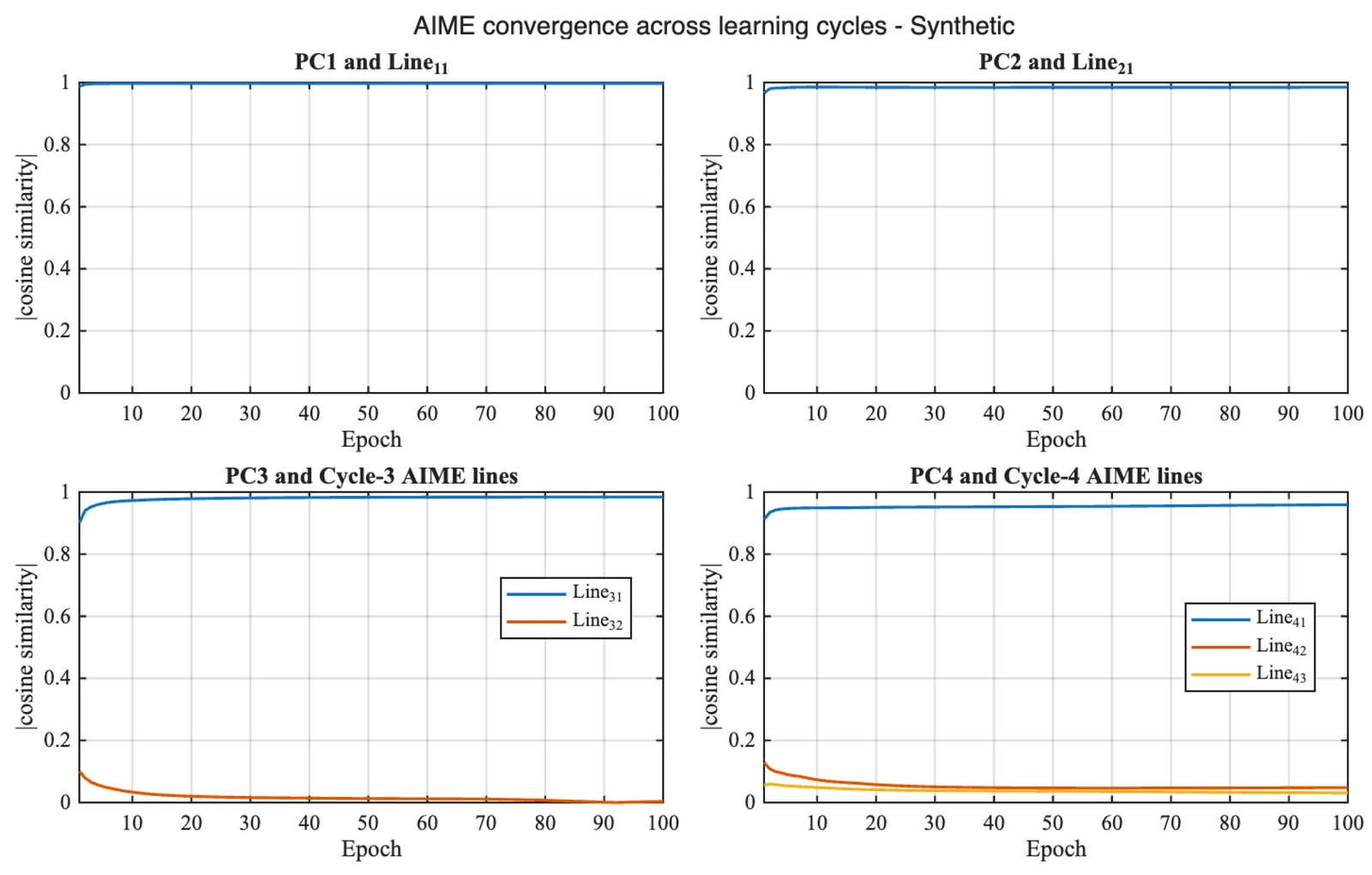
Convergence of AIME learning across successive cycles on the synthetic dataset. For clarity, the first 100 learning epochs are shown. Each panel reports the absolute cosine similarity between representative neural weight vectors associated with the final AIME components and the corresponding principal component. Overall, convergence is achieved faster than in Iris across all cycles. In cycles 1 and 2, the recovered AIME directions remain strongly aligned with PC1 and PC2, respectively. In cycles 3 and 4, multiple AIME components emerge: one representative trajectory remains strongly aligned with the corresponding principal component, whereas the additional branches progressively become nearly orthogonal to that principal direction. The results illustrate both the convergence of PC-aligned AIME components and the emergence of additional directional components under the more dispersed variance structure of the synthetic data.

Figure 8 and Figure 9 show that the representative weight vectors progressively increase their directional alignment with the corresponding principal components over successive learning epochs for both datasets. The convergence trajectories therefore complement the post-hoc component visualizations in Figure 6 and Figure 7, showing that the final alignment between AIME components and principal directions emerges progressively through the AIME learning process rather than only appearing in the final learned-weight configuration.

Numerical outcomes: Table 4 and Table 5 show the correlations between the AIME components and true PCs, and the explained variances by the AIME components. Table 6 and Table 7 further summarize the multi-seed reproducibility analyses, reporting the mean, standard deviation, and 95% confidence interval of the AIME–PC alignment across 100 independent random seeds. Table 8 reports the additional Wine and WDBC benchmark results, comparing the alignment of AIME, Oja, and Sanger’s GHA components with the first three principal components.

**Figure 10:**
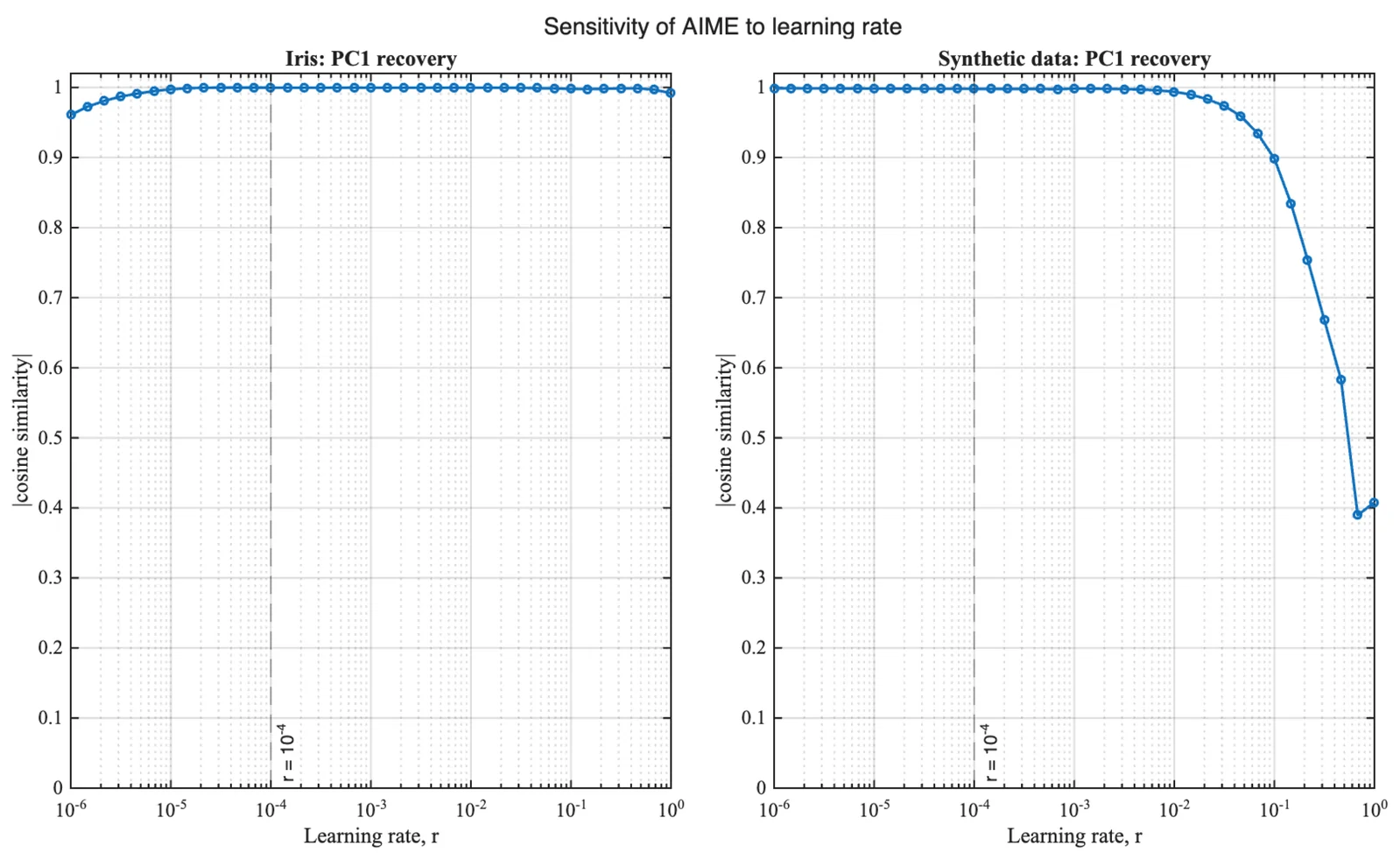
Learning-rate sensitivity of AIME for PC1 recovery on the Iris and synthetic datasets. The learning rate *r* was varied logarithmically from $10^{-6}$ to 1 while all other implementation settings were held fixed. The vertical dashed line indicates the learning rate $r=10^{-4}$ used in the primary simulations. AIME maintains strong PC1 alignment across a broad range of learning rates for both datasets, with the synthetic-data alignment decreasing only at substantially larger learning rates. The selected value $r=10^{-4}$ therefore lies within a broad stable regime rather than representing a narrowly tuned optimum.

For sensitivity analysis, as shown in Figure 10, AIME maintains high PC1 alignment over a broad range of learning rates. In particular, the manuscript setting of $r=10^{-4}$ lies within a wide stable regime rather than at a narrowly tuned optimum. For the synthetic dataset, alignment begins to deteriorate only at substantially larger learning rates, where individual updates become correspondingly more aggressive. These results indicate that the reported PC1 recovery is not sensitive to small variations around the selected learning rate.

## 7. Related Work

We show that specific biological characteristics of a single cortical circuit can give rise to two distinct and well-recognized computational behaviors: hierarchical clustering and component-based feature extraction. Rather than deriving a closed-form mathematical equivalence between these operations, we formalize how both behaviors emerge from the same underlying circuit dynamics under different operating regimes.

This perspective suggests a unifying computational framework, in which clustering and component analysis arise as limiting cases of a shared biological mechanism. In this section, we review related work in machine learning and computational neuroscience that has explored connections between these two classes of algorithms

### 7.1. Biologically Inspired Precedents to LI-HC

Biologically inspired clustering mechanisms have been extensively studied in computational neuroscience. Early work by von der Malsburg [11] proposed that lateral inhibition in the visual cortex could support self-organized clustering. This idea was further developed by Grossberg [52], who incorporated memory dynamics, and by Kohonen [53], who introduced competitive learning in self-organizing maps (SOMs). Rumelhart and Zipser [54] later explored competitive networks for unsupervised feature discovery. These foundational studies influenced subsequent biologically motivated models of clustering (e.g., [55,56]).

Rodriguez et al. [30] presented a computational model of thalamocortical circuitry that performs clustering and hierarchical clustering, providing a concrete implementation of these principles. In this context, our work is related in that it examines competitive dynamics within a biologically grounded circuit architecture (L-FLIC), while placing additional emphasis on how such dynamics can give rise to structured computational behavior.

Motivated by this line of work, we further investigate the role of cooperative dynamics within the same circuit framework, leading to the development of AIME, which captures complementary aspects of data structure in terms of spatial dispersion.

### 7.2. PCA with Biological Plausibility

A number of neural network algorithms for PCA exhibit strong biological plausibility. Hebbian learning, one of the most fundamental and widely studied neurophysiological learning rules, is known to perform PCA through incremental updates ([57]). Oja’s rule [16] refines Hebbian learning by introducing a normalization term to prevent unbounded growth of synaptic weights. Building on this foundation, the Bienenstock–Cooper–Munro (BCM) rule introduces a dynamic threshold that governs whether synaptic strength increases (long-term potentiation, LTP) or decreases (long-term depression, LTD), with the threshold adapting over time according to the average level of postsynaptic activity [58]. Extending these ideas to multi-output networks, Sanger [51] developed the Generalized Hebbian Algorithm (GHA), enabling the extraction of multiple principal components. Despite differences in biological fidelity and implementation, these algorithms share a common mechanism: updates are driven by the product of presynaptic and postsynaptic activity, and the resulting dynamics implicitly approximate a decomposition of the input covariance structure.

More recently, Pehlevan and Chklovskii [59] extended Hebbian/anti-Hebbian networks for dimensionality reduction by embedding them within a similarity-matching objective, formulating learning as a minimax interaction between feedforward and lateral connections. Their framework generalizes to tasks such as independent component analysis (ICA), clustering, and manifold tiling, while maintaining biological plausibility through local learning rules and sequential inputs.

In contrast to these architectures, the proposed AIME algorithm differs in two key respects. First, AIME follows an indirect training strategy: it does not explicitly construct a covariance matrix, perform eigen-decomposition, or optimize a predefined objective via backpropagation. By comparison, Hebbian-style algorithms implicitly approximate the input covariance structure, as the product of input and output activities yields a scaled estimate of covariance. Second, AIME allows excitatory neurons to access input signals independently, rather than relying on the co-activation principle central to Hebbian learning, where synaptic modification depends on simultaneous pre- and postsynaptic activity.

Overall, AIME represents a complementary perspective, in which PCA-like behavior emerges from circuit-level dynamics rather than explicit covariance-based learning mechanisms.

### 7.3. PCA with Competitive Learning

The AIME algorithm is related to competitive learning frameworks. The use of competitive learning for PCA has been explored in prior work on self-organizing maps (SOMs), originally proposed by Kohonen [53] for categorization tasks.

Ritter et al. [60] extended Kohonen’s SOM to compute principal curves and surfaces, which can be viewed as nonlinear generalizations of PCA. For data with approximately ellipsoidal structure, principal components arise as a special case of principal curves. Through competitive learning, their method produces smooth curves or surfaces that pass through the central region of the data distribution. The learning process updates winning weight vectors toward the local center of gravity of the input space.

Kohonen [61,62] subsequently introduced the Adaptive Subspace Self-Organizing Map (ASSOM) as an alternative approach to PCA. ASSOM employs competitive learning to adapt a set of subspace-based representations associated with temporal input episodes. In this model, a “winning” subspace is selected for each episode (a set of input vectors sampled over a temporal window) and is updated across the samples within that episode. The output of each neuron reflects the degree of alignment between the input data and the corresponding learned subspace.

López-Rubio et al. [63] later proposed Principal Components Analysis with Competitive Learning (PCACL), a neural approach that improves upon traditional local PCA methods. In PCACL, the winning neuron is selected based on projection error with respect to the leading eigenvectors of local covariance estimates. The model updates weights toward local means and covariance structures, enabling efficient dimensionality reduction while preserving key properties of PCA.

### 7.4. Synergistic Connections Between PCA and Clustering

Over the past two decades, numerous studies have explored the close relationship between principal component analysis (PCA) and clustering from mathematical, algorithmic, and application-oriented perspectives.

From a theoretical standpoint, Ding and He [31] established a fundamental connection between PCA and *K*-means clustering by showing that the cluster centroid subspace is closely approximated by the leading principal subspace. Shortly afterward, Zass and Shashua [64] provided a unified algebraic framework in which spectral clustering, kernel *K*-means, and related methods were interpreted through completely positive matrix factorization. Bauckhage [65] further demonstrated that the conventional *K*-means objective itself can be written as a constrained matrix factorization problem, reinforcing the close mathematical relationship between clustering and low-rank representations. More broadly, Mairal et al. [66] showed that sparse coding, dictionary learning, non-negative matrix factorization, and sparse PCA can all be formulated within a unified matrix factorization framework.

Building upon these theoretical foundations, many studies have incorporated PCA directly into clustering algorithms. Dayal [32] proposed a hierarchical divisive clustering method based on successive principal directions. Honda et al. [33] extended PCA-guided *K*-means through adaptive variable weighting, while Tajunisha and Saravanan [36] employed PCA during cluster initialization to improve clustering stability and computational efficiency. More recently, Chen et al. [40] integrated PCA embedding directly into the optimization objective of fuzzy C-means clustering. Other studies have further extended PCA-guided clustering to cluster validation, mixed-type data, and application-specific scenarios.

More recently, PCA has also been investigated as a clustering mechanism in its own right. Mukherjee and Zhang [39] demonstrated theoretically that PCA compression itself enhances cluster separability by reducing intra-cluster variation, suggesting that PCA alone already possesses substantial clustering capability. Li et al. [67] further incorporated this compression-ratio principle into a confidence-based clustering framework for single-cell RNA sequencing analysis.

Collectively, these studies demonstrate that PCA and clustering are closely related mathematically and algorithmically, and can often be combined in complementary ways. In contrast, our work approaches this relationship from a different perspective. Rather than explicitly integrating PCA into clustering algorithms (or vice versa), we propose that both clustering behavior and component-based representations emerge naturally from the same biologically inspired neural circuit operating under different inhibitory regimes. This provides a unified mechanism-driven interpretation that complements the existing mathematical literature.

## 8. Conclusions and Discussion

### 8.1. AIME as the Dual of LI-HC

In this work, we present two computational operations—LI-HC and AIME—that arise from the same biologically grounded architecture, the Local Feedback Inhibitory Circuit (L-FLIC) within the thalamocortical system. Both operations share an identical circuit structure (Figure 1), differing only in a single parameter: the strength of lateral inhibition.

When inhibition is strong, the circuit exhibits competitive dynamics, resulting in LI-HC. When inhibition is weak or negligible, the same circuit transitions to a cooperative regime, giving rise to AIME. Despite this minimal change in parameterization, the resulting computational behaviors are functionally distinct.

This observation suggests a conceptual duality between clustering and component-based dimensionality reduction: two different statistical operations emerging from a shared circuit mechanism under different operating regimes. Rather than establishing a formal mathematical equivalence, our results highlight a mechanistic connection grounded in circuit dynamics.

Shared foundations

The duality between AIME and LI-HC originates at the structural level. Both algorithms are derived from the same circuit architecture (L-FLIC), with identical connectivity and synaptic organization. This shared substrate enables multiple computational roles to emerge from a single neural system.

In addition, both algorithms rely on similar local learning dynamics: weight updates are driven by activity-dependent interactions and proceed iteratively over time. This common update structure reflects a shared computational foundation, despite differences in emergent behavior.

Key differences

The distinction between LI-HC and AIME arises from their operating regimes. LI-HC enforces strong competition through lateral inhibition, leading to winner-take-all clustering behavior. In contrast, AIME operates in a regime where inhibition is reduced, resulting in cooperative dynamics. This shift leads to several key differences:

1. Input representation. AIME operates directly on the raw input space without normalization, preserving the natural dispersion of the data. In contrast, LI-HC normalizes inputs, emphasizing relative similarity for clustering.
2. Neuronal interaction. In the absence of strong lateral inhibition, AIME allows neurons to respond independently to input signals, whereas LI-HC enforces competition, leading to localized specialization.
3. Representation objective. LI-HC produces discrete cluster assignments, while AIME identifies dominant directions (axes) of data variation, resulting in a continuous representation of data structure.

### 8.2. Computational Efficiency and Scalability

We furthermore evaluate the time and space complexity of AIME and LI-HC, alongside their traditional counterparts—such as divisive hierarchical clustering and PCA—and assess their scalability.

Time and space complexity: Given *n* data points of dimension *p*, let *i* be the average number of iterations per hierarchy and *q* the total number of neural weights. Suppose the goal is to obtain *m* final clusters in hierarchical clustering, with an average branching factor of *B*. The time complexity of LI-HC is O(npiq·logn), while for AIME, it is $O(np^{2}iq)$. The space complexity for both LI-HC and AIME is O(np+qp) as all weight updates occur in place.

For traditional divisive hierarchical clustering (DHC) with each cycle as a plain K-Means, the time complexity is O(npiB·logn), where logn represents the maximum depth of the hierarchy. The space complexity for DHC is $O\left(np+\frac{mB}{B-1}p\right)$. The second part represents the total number of intermediate units in the hierarchy [68].

The regular procedure of PCA has time complexity of $O(np^{2}+p^{3})$, which can be further simplified as $O(p^{3})$. It is combined with $O(np^{2})$ for computing the covariance matrix and $O(p^{3})$ for eigen-decomposition. The regular procedure of PCA has a total space complexity of $O(np+p^{2}+pk+nk)$, where each term corresponds to a key component in the algorithm. The term O(np) accounts for storing the centered input data matrix with *n* samples and *d* features. The $O(p^{2})$ term arises from forming the p×p covariance matrix. The O(pk) component reflects the storage of the top *k* eigenvectors (i.e., the principal components), each of dimensionality *p*. Finally, O(nk) represents the memory required to store the projected data in the *k*-dimensional PCA space. Depending on the relative values of *n* and *p*, either the original data or its covariance matrix dominates the memory usage, since k≪p.

Scalability: Based on Table 9, all algorithms exhibit increasing complexity as the input scale grows. For a fixed number of samples *n*, as the dimensionality *p* increases, the time complexity of clustering increases linearly, whereas that of PCA or AIME grows at least quadratically. Notably, when *p* becomes significantly large, the cubic term in traditional PCA leads to substantial computational overhead. In terms of space complexity, all methods scale linearly with input size, except for traditional PCA, which requires extensive memory to store the full covariance matrix.

It is worth noting that increasing the dimensionality of the input data can collaterally affect the number of iterative steps and may lead to greater sensitivity to other hyperparameters. This underscores more computational efforts in hyperparameter tuning. We tested a one-cycle run of AIME on a 10,000×784 subset of the *Modified National Institute of Standards and Technology* (MNIST) database image dataset [48]. The result produced two lines that explained the input variation comparably well, one of which closely aligned with the true first principal component. In this experiment, we used a significantly smaller learning rate ($10^{-7}$, compared to $10^{-4}$ used for the Iris dataset) to achieve acceptable performance; the results are shown in Table 10.

As an additional comparison, we conducted a one-cycle (single-hierarchy) experiment on the same MNIST dataset to evaluate the performance of the LI-HC algorithm relative to a traditional Lloyd’s K-means clustering algorithm [45].

The LI-HC algorithm identified 11 clusters (note that the number of clusters is not expected to match the number of ground truth labels, given the unsupervised nature of the method). Accordingly, we set K=11 for K-means clustering to ensure a fair comparison. Table 11 presents standard clustering metrics [69] for both methods. The results show that LI-HC and K-means achieve comparable clustering performance, thereby supporting the validity of LI-HC.

It is important to note that our goal is not to develop a method that consistently outperforms existing benchmarks, but to introduce an alternative clustering approach with biologically inspired capabilities that broadens the methodological landscape. Whether one chooses LI-HC or K-means ultimately depends on the user’s goals and preferences. Notably, LI-HC has the advantage of not requiring the number of clusters *K* to be specified in advance, which can significantly reduce the time and effort typically spent on hyperparameter tuning. However, as indicated by the computational complexity, there exists a trade-off: while K-means requires explicit tuning of *K*, LI-HC involves the selection and management of neural weight vectors. This trade-off introduces a new dimension of flexibility, allowing users to choose the approach that best aligns with their computational constraints and conceptual framing of the data.

### 8.3. AIME as a Valuable Alternative to Traditional PCA

AIME offers a biologically motivated alternative to traditional PCA, with potential advantages in interpretability, flexibility, and computational structure. In particular, AIME may be advantageous in the following scenarios:

1. PCA compatibility and computational efficiency. AIME produces components that capture major directions of data dispersion, and in many cases exhibits behavior comparable to PCA. Its iterative weight-update mechanism avoids explicit construction and decomposition of a covariance matrix, and scales linearly with the dimensionality *p*. This makes AIME particularly appealing in high-dimensional settings where classical PCA may be computationally or memory intensive.
2. Interpretability diversity. While PCA identifies a single orthogonal basis ordered by variance, its representation may be less informative when multiple directions exhibit comparable variance. In such cases, AIME can yield multiple meaningful directions that reflect alternative structures in the data. This can provide additional interpretability in domain-specific contexts—for example, revealing sector-level patterns in financial data or biologically relevant groupings in genomics.
3. Robustness through redundancy. In settings where principal components are sensitive to noise or distributional shifts, AIME’s ability to produce multiple candidate directions spanning similar subspaces may offer increased robustness. These alternative components can serve as complementary representations, potentially stabilizing downstream analyses.

While AIME exhibits these practical advantages, the primary objective of this work is not to replace existing PCA methodologies, but to investigate what forms of computation emerge from biologically grounded circuit constraints. Under such constraints, one might expect tradeoffs in efficiency or precision. It is therefore notable that the resulting algorithms achieve performance that is comparable to established methods in many settings.

More broadly, this suggests that biologically constrained systems may give rise to computational strategies that are both efficient and adaptable. Understanding these emergent mechanisms may provide useful insights for the design of future machine learning models.

### 8.4. Challenges and Future Directions

There are several fertile directions for future research:

- Extension to non-linear structure. The current formulation of AIME is well-suited for capturing linear structure in the data, but does not directly address non-linear manifolds. Extending AIME to handle non-linear representations remains an important open direction.
- Multiplicity of components per cycle. Empirical observations indicate that each learning cycle yields a limited number of AIME components (often no more than two). The underlying reason for this constraint is not yet fully understood and warrants further theoretical investigation.
- Component identification and clustering. The current approach relies on K-Means clustering to identify AIME components from learned weight vectors, with the number of clusters selected empirically. More principled alternatives may improve robustness. In particular, the clustering module of LI-HC provides a potential mechanism for automatically determining component groupings. Preliminary implementations of this approach exist, but are not explored in detail here.
- Theoretical and empirical refinement. While this work combines analytical insights with empirical validation, further development is needed to strengthen the theoretical foundations and to evaluate performance across a broader range of datasets and conditions.
  At present, these directions remain exploratory, and we do not yet provide a concrete algorithmic framework or empirical validation. Establishing such methods and evaluating their practical utility constitutes an important direction for future work.

## Figures and Tables

**Table 1:** Clustering evaluation metrics for LI-HC and hierarchical Lloyd *K*-means on the synthetic dataset across three hierarchical levels. The first, second, and third cycles correspond to 4, 8, and 24 clusters, respectively, and each level is evaluated against its corresponding ground-truth labels. Both methods recover the same hierarchical partitions on this deliberately well-separated dataset, resulting in identical external and internal clustering metrics at the reported precision. These results should be interpreted as a controlled validation of LI-HC on the constructed hierarchy rather than as evidence of general equivalence between LI-HC and *K*-means.

Method	Cycle	Purity	NMI	RI	SC	CHI	DBI
LI-HC	1	1.000	1.000	1.000	0.98329	38,139	0.18665
LI-HC	2	1.000	1.000	1.000	0.97063	$2.9762\times 10^{5}$	0.24335
LI-HC	3	1.000	1.000	1.000	0.93803	$1.0834\times 10^{6}$	0.36124
K-means	1	1.000	1.000	1.000	0.98329	38,139	0.18665
K-means	2	1.000	1.000	1.000	0.97063	$2.9762\times 10^{5}$	0.24335
K-means	3	1.000	1.000	1.000	0.93803	$1.0834\times 10^{6}$	0.36124

**Table 2:** Explained variances of both the Iris data and the synthetic data. Two sub-tables display the first seven principal components of the data (if applicable) both individually and cumulatively. PCs of the Iris data show steep gradients, with PC1 explaining 92.2% of the variance and the following PCs explaining variances three times greater than the next PC. Whereas in the synthetic data, the explained variances progressively decrease through principal components. Notably, the synthetic data’s fourth and fifth PCs explain nearly identical variance percentages, indicating that the data spreads more evenly along these two axes. N/A indicates that no additional principal components exist for the four-dimensional Iris dataset.

**(a) Explained variances, individual**								
**Data**	**PC1**	**PC2**	**PC3**	**PC4**	**PC5**	**PC6**	**PC7**	**…**
Iris data	92.2%	5.4%	1.8%	0.5%	N/A	N/A	N/A	N/A
Synthetic data	31.5%	12.9%	8.2%	6.8%	6.1%	4.5%	2.9%	…
(**b**) **Explained variances, cumulative**								
**Data**	**PC1**	**PC2**	**PC3**	**PC4**	**PC5**	**PC6**	**PC7**	**…**
Iris data	92.2%	97.6%	99.5%	100%	N/A	N/A	N/A	N/A
Synthetic data	31.5%	44.4%	52.6%	59.4%	65.5%	70.0%	72.9%	…

**Table 3:** Implementation settings used in the reported AIME simulations. The number of weight vectors is a population-sampling choice rather than a minimum requirement of the AIME algorithm. K-Means is applied only after AIME training to summarize groups of converged weight vectors.

Dataset	Learning Rate	Weight Vectors	Epochs	Primary Run Seed	Post-Hoc K
Iris	$10^{-4}$	600	400/cycle	1	2
Synthetic	$10^{-4}$	600	400/cycle	1	2 or 4
MNIST	$10^{-7}$	70	500	1	4

**Table 4:** Numerical outcomes of AIME on the Iris dataset after four successive learning cycles. (**a**) shows the absolute cosine similarities between the AIME component recovered in each cycle and the four true principal components. The highest value in each row is highlighted in red and occurs for the corresponding principal component, with all four values close to 1, indicating strong directional alignment. In (**b**), the cumulative explained variances obtained from the AIME components closely match those of the true principal components, indicating that the recovered directions capture nearly the same data dispersion.

(**a**) **Absolute Correlations**				
	**PC1**	**PC2**	**PC3**	**PC4**
Cycle 1 component	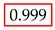	0.024	0.010	0.002
Cycle 2 component	0.025	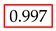	0.023	0.063
Cycle 3 component	0.002	0.022	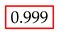	0.053
Cycle 4 component	0.012	0.035	0.019	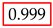
(**b**) **Cumulative Explained Variances**				
	**First**	**Second**	**Third**	**Fourth**
True PC	92.2%	97.6%	99.5%	100%
AIME Components	92.4%	97.7%	99.5%	100%

**Table 5:** Numerical outcomes of AIME on the synthetic dataset after four learning cycles. Panel (**a**) reports the absolute cosine similarities between the recovered AIME components and the first few principal components (PCs). The red boxes highlight the cosine similarities between the recovered components and their corresponding true PCs. A single component is recovered in cycles 1 and 2, whereas two components (A and B) emerge in cycle 3 and three descendant components (A, B, and C) are obtained in cycle 4, following the branching structure shown in Figure 5. The green rows denote the branch originating from Cycle 3 component A, which yields Cycle 4 components A and B, while the blue rows denote the branch originating from Cycle 3 component B, which yields Cycle 4 component C. Panel (**b**) compares the cumulative explained variances of the three resulting learning trajectories with those of the first four true PCs, normalized within the selected component subset. Because the Cycle 1 and Cycle 2 components are shared by all three trajectories, only the distinguishing Cycle 3–Cycle 4 branches are listed. Despite differences in directional alignment, the three trajectories capture comparable cumulative variance, consistent with the more evenly distributed variance structure of the synthetic dataset. N/A indicates that no additional principal components exist for the four-dimensional Iris dataset.

(**a**) **Absolute Correlations**						
	**PC1**	**PC2**	**PC3**	**PC4**	**PC5**	**…**
Cycle 1 component	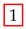	0.03	0.01	0.01	0.02	…
Cycle 2 component	0.03	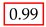	0.08	0.01	0.01	…
Cycle 3 component A	0.02	0.09	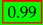	0.01	0.12	…
Cycle 3 component B	0	0.02	0.04	0.96	0.28	…
Cycle 4 component A	0	0.02	0.03	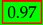	0.23	…
Cycle 4 component B	0.02	0.01	0.12	0.04	0.98	…
Cycle 4 component C	0.02	0.09	0.99	0.01	0.13	…
(**b**) **Cumulative Explained Variances**						
	**First**	**Second**	**Third**	**Fourth**	**…**	
original true PCs	31.5%	44.4%	52.6%	59.4%	…	
normalized true PCs	53.0%	74.7%	88.5%	100%	N/A	
Cycle 3 A → Cycle 4 A	53.1%	74.7%	88.6%	100%	N/A	
Cycle 3 A → Cycle 4 B	53.7%	75.5%	89.5%	99.9%	N/A	
Cycle 3 B → Cycle 4 C	53.1%	74.7%	86.1%	100%	N/A	

**Table 6:** Multi-seed reproducibility of AIME on the Iris dataset. Values summarize the absolute cosine similarity between the AIME component and the corresponding principal component across 100 independent random seeds for each learning cycle.

Cycle	Mean	SD	95% CI Lower	95% CI Upper
1	0.99966	$1.40\times 10^{-6}$	0.99966	0.99966
2	0.99774	$2.19\times 10^{-5}$	0.99774	0.99775
3	0.99875	$7.41\times 10^{-5}$	0.99874	0.99877
4	0.99716	$4.42\times 10^{-5}$	0.99715	0.99717

**Table 7:** Multi-seed reproducibility of AIME on the synthetic dataset. Values summarize the absolute cosine similarity between the best PC-aligned AIME component and the corresponding principal component across 100 independent random seeds for each learning cycle.

Cycle	Mean	SD	95% CI Lower	95% CI Upper
1	0.99818	$2.07\times 10^{-4}$	0.99814	0.99822
2	0.99148	$1.66\times 10^{-3}$	0.99115	0.99181
3	0.98156	$8.33\times 10^{-3}$	0.97990	0.98321
4	0.97587	$1.10\times 10^{-2}$	0.97369	0.97805

**Table 8:** Additional benchmark comparison of AIME, Oja’s learning rule, and Sanger’s Generalized Hebbian Algorithm (GHA) on the Wine and WDBC datasets. Values report the absolute cosine similarity between each recovered component and the corresponding principal-component direction obtained by standard PCA. For AIME, when multiple components emerged within a learning cycle, the component with the strongest alignment to the corresponding principal component was reported. Oja’s rule and Sanger’s GHA, both explicitly formulated for neural principal-component extraction, exhibit near-exact correspondence with the PCA reference directions. AIME instead estimates principal directions through a distinct learning mechanism and therefore is not expected to reproduce PCA exactly. Nevertheless, consistently high AIME–PC alignment is observed across both datasets. The moderate decrease in AIME alignment for later components is consistent with the sequential learning procedure, in which removal of the dominant variance directions leaves progressively weaker directional structure for subsequent estimation.

Dataset	Method	PC1	PC2	PC3
Wine	AIME	0.9969	0.9904	0.9576
	Oja	0.9999	1.0000	1.0000
	Sanger GHA	0.9999	1.0000	1.0000
WDBC	AIME	0.9978	0.9894	0.9218
	Oja	0.9959	0.9968	0.9973
	Sanger GHA	0.9968	0.9987	0.9979

**Table 9:** Comparison of time and space complexity.

	LI-HC	DHC	AIME	PCA
Time	O(npiq·logn)	O(npiB·logn)	$O(np^{2}iq)$	$O(p^{3})$
Space	O(np+qp)	$O\left(np+\frac{mB}{B-1}p\right)$	O(np+qp)	$O(np+p^{2}+pk+nk)$

**Table 10:** One-cycle AIME with MNIST dataset. We obtain two lines (plus the true first PC as a benchmark) from AIME, with Line 2 more closely aligned with the true PC1 obtained from standard PCA. We project the mean-centered input data matrix *X* onto each line and compute the variance of the resulting 1D projection. The explained variance for a direction *u* is calculated as $\mathrm{Var}\left(Xu\right)/\sum_{i=1}^{3}\mathrm{Var}\left(Xu_{i}\right)$, where $u_{i}\in \left\{\mathrm{PC}1,\mathrm{Line}\;1,\mathrm{Line}\;2\right\}$. This yields the relative variance contribution of each line with respect to the total variance explained by all three. It is important to note that the explained variances in this table are calculated using a different method than those in previous tables. Importantly, all reported directions capture a similar magnitude of variance in the input data.

	Corr with PC1	Expl. Var
Line 1	0.76	31%
Line 2	0.96	34%
PC 1	1.0	35%

**Table 11:** One-hierarchy LI-HC vs. K-means on the MNIST dataset. Both external metrics—Purity, Normalized Mutual Information (NMI), and Rand Index—and internal metrics—Silhouette Coefficient (SC), Calinski-Harabasz Index (CHI), and Davies-Bouldin Index (DBI)—are used to evaluate clustering performance, measuring alignment with true labels and intrinsic cluster cohesion, respectively. In scenarios with high cluster separability (as in Table 1), LI-HC and K-means achieve near-perfect scores across all metrics. However, when applied to the MNIST dataset—known for its high dimensionality and relatively low inherent separability—both LI-HC and K-means yield moderate, yet reasonable, performance. Despite this, LI-HC and Lloyd’s standard K-means algorithm still produce highly similar results across all metrics on MNIST, suggesting that the two methods generate comparable cluster assignments. This strong agreement with a well-established baseline further validates the reliability and effectiveness of LI-HC.

Metrics	Purity	NMI	Rand Index	SC	CHI	DBI
LI-HC on MNIST	0.6	0.5	0.9	0.1	369.7	2.8
K-means on MNIST (K = 11)	0.6	0.5	0.9	0.1	368.5	2.9

**Figure A1:**
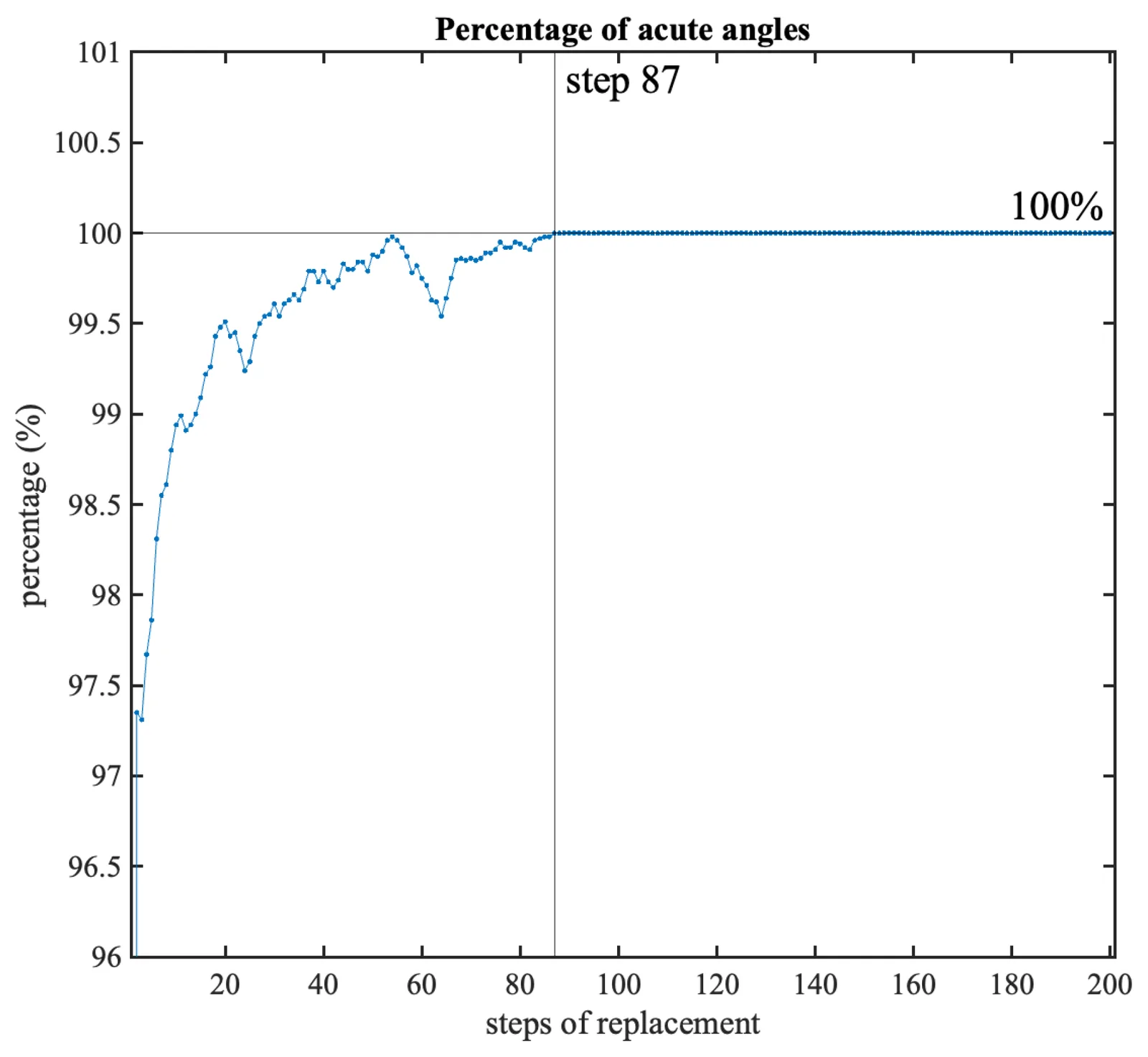
Empirical behavior of the acute-partition finding heuristic. At each replacement step, Algorithm 3 determines a new antipodal selection $\tilde{X}$ and recomputes its mean vector. Each point represents the percentage of vectors in $\tilde{X}$ that form acute angles with the current mean. In the illustrated simulation, the percentage reaches and remains at 100%, indicating that a stable acute partition has been identified.

**Figure A2:**
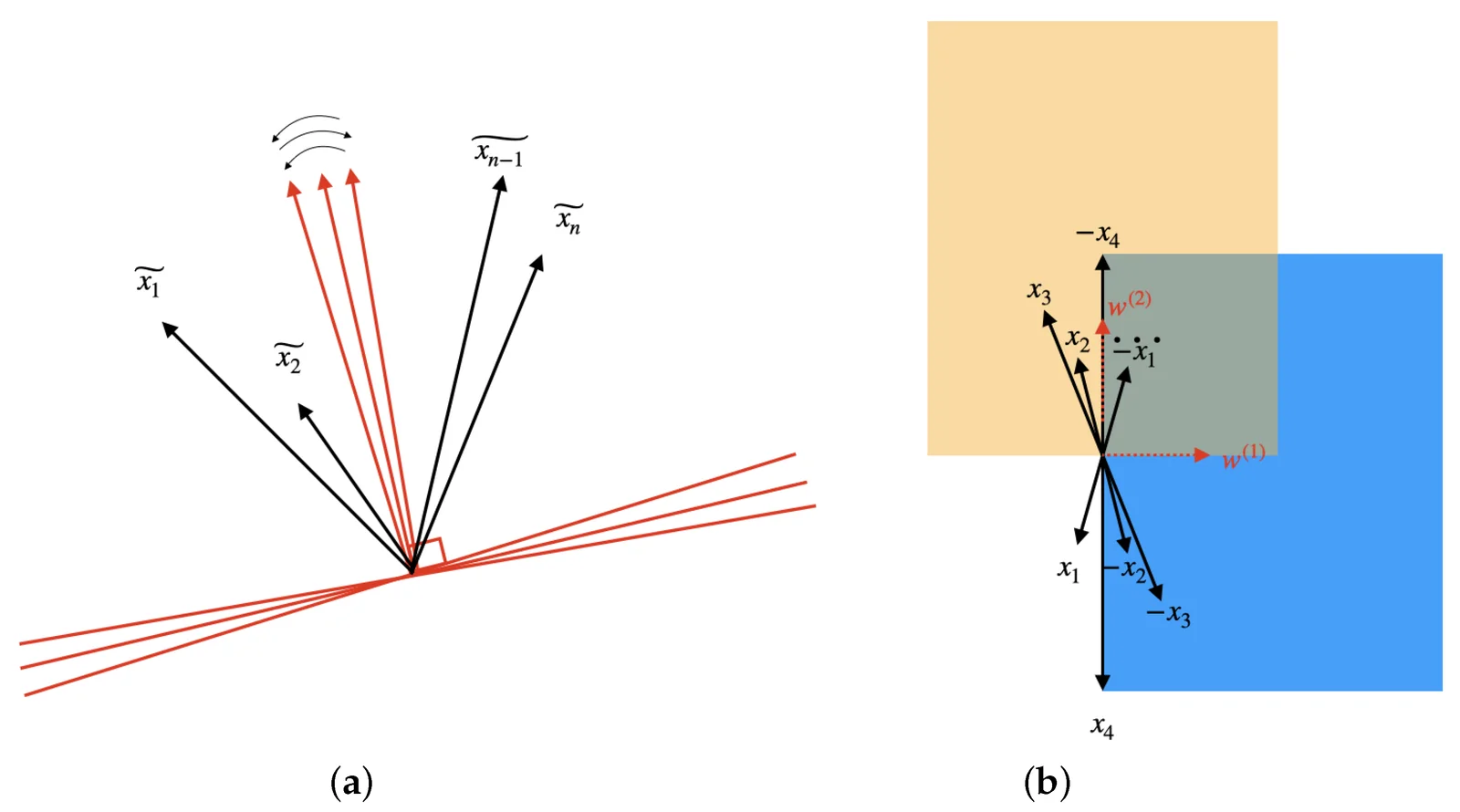
Illustration of the convergence reasoning for a single AIME neuron. (**a**) After the antipodal selection becomes stable, subsequent weight updates produce only small fluctuations around the acute-partition mean without changing the selected vectors. (**b**) Comparison of two candidate acute partitions. The blue region represents a weaker partition, where small perturbations of the weight vector can change the antipodal selection and therefore alter the partition. The yellow region represents a stronger acute partition whose selected vectors remain stable under comparable perturbations, making convergence toward this partition more likely.

## Data Availability

All data and code required to reproduce the results of this study are publicly available at https://github.com/charlesl3/LFLIC (accessed on 20 August 2026). The study utilized synthetic datasets generated in MATLAB R2026a, built-in MATLAB datasets, and a publicly available synthetic dataset from Statistics Globe.

## Appendix A

### Appendix A.1. The Concepts Underlying Iterative Masking

The concepts underlying iterative masking are solidly in the literature, relying on two specific facts: (i) the nucleus reticularis layer of the thalamus is very well known to topographically inhibit the thalamic structures it overlays, and (ii) the corticothalamic system is very well known to iteratively sample, such as multiple timed saccades in vision (e.g., Melcher et al. [70]), even rhythmic repeated firing in audition (e.g., L’Hermite and Zoefel [71]; Kayser [72]). Moreover, the inhibitory cell type in nucleus reticularis is well known to have a very long inhibitory period (Zhang and Jones [73]), lasting substantially longer than the repeated sampling. Thus each successive sample incontrovertibly receives different input information, ‘masked’, i.e., selectively inhibited, by this long-duration nucleus reticularis inhibition.

Moreover, the full literal operation of iterative masking has itself been long cited in the literature by the present authors (Rodriguez and Granger [74]) and also by many other researchers (e.g., George and Hawkins [75]; Treves [76]; Jeon [77]).

The question of successive iterations erasing previous information is an extremely good one, which also has been extensively studied (e.g., Rodriguez et al. [30]; Rodriguez and Granger [74]). An initial intuition is helpful here: multiple variants of an image (e.g., several different flowers) are similar literally by virtue of being near each other in the input space; thus each image has a shared core $\vec{x}$ and some variation $\vec{v}$, so that every image is $\vec{x}$ + $\vec{v}$ with the same $\vec{x}$ and different $\vec{v}$ for each image. Over multiple exposures, simple unsupervised clustering occurs in a very broad range of biological algorithms, simply by Hebbian incremental strengthening of the shared parts of the many flower images which are incremented far more than the unshared portions. Thus the single cluster for all of these flower images points roughly in the direction $\vec{x}$ with each specific image offset from that centroid according to the extra $\vec{v}$ component. The initial network response to any of the flower images is $\vec{x}$. Thus the feedback inhibition also corresponds to $\vec{x}$, inhibiting or ‘masking’ that out of the input in time for the next sampling (e.g., saccade). The resulting next sample therefore is the image minus the feedback, i.e., $\vec{x}$ + $\vec{v}-\vec{x}$ = $\vec{v}$.

Now the question arises of whether that new ‘masked’ (partially inhibited) input will interfere with or erase previous information. It is notable that the direction of $\vec{v}$ is unconstrained with respect to the direction of $\vec{x}$. Since these are high dimensional vectors, there is a very low likelihood that the direction of $\vec{v}$ is (accidentally) similar to the direction of $\vec{x}$. In the rare case that it is, there will indeed be interference, and such cases could be tested as future research. Much more commonly, the direction of $\vec{v}$ will be dissimilar to that of $\vec{x}$, in which case the new $\vec{v}$ input will selectively “win” on target excitatory cells that do not overlap with those that respond to $\vec{x}$.

### Appendix A.2. Reasoning of Convergence: *w*_*i*+1_ = *w_i_* + *r* ·(*x* − *w_i_*)

Fixed-point formulation. In this section, we revisit the competitive learning rule through a numerical methods perspective, providing a valid justification of its convergence. Specifically, we utilize fixed-point iteration principles ([78]) to formally establish that a single initial weight vector $w_{0}$, updated through $w_{i+1}=w_{i}+r\cdot \left(x-w_{i}\right)$, converges towards a single input vector *x*, where $w_{i}$ indicates $w_{0}$ has been updated for *i* steps. Consider the real-valued fixed-point equation w=g(w), where g(w)=w+r·(x−w); it can be either scalar or vectorized. A fixed point of this mapping satisfies w=g(w), which is equivalent to r·(x−w)=0, i.e., x=w, where the learning rate 0<r<1. Such a formulation indicates that the competitive learning rule, $g\left(w_{i}\right)=w_{i}+r\cdot \left(x-w_{i}\right)$, is an updating equation of fixed-point iteration, through which the function $f\left(w_{i}\right)=r\cdot \left(x-w_{i}\right)$ can find its root. We denote the fixed-point problem in the form below:

(A1) $$\left\{\begin{array}{c} g\left(w_{i}\right):=w_{i}+r\cdot \left(x-w_{i}\right)\\ w_{i+1}=g\left(w_{i}\right)\\ w_{n}=g\left(w_{n}\right) \end{array}\right.$$

where *r* represents the learning rate and $w_{n}$ is the fixed point such that $g\left(w_{n}\right)=w_{n}$. Before investigating the convergence of the competitive learning rule, we briefly introduce the conditions of the existence and uniqueness of the fixed point in generality with two theorems.

**Theorem** **A1** (existence of fixed point). *Let g(w) be any continuous and real-valued function on a closed interval [a,b] and differentiable on (a,b), and ∀w∈[a,b], g(x)∈[a,b]. Then w=g(w) has at least one solution $w_{n}$ in [a,b]; i.e., g(w) has at least one fixed point $w_{n}$ on [a,b].*

**Proof.** Define f(w)=w−g(w) (also continuous and real-valued) and we know that f(a)≤0,f(b)≥0 from the conditions. By the existence theorem of the zero point from real analysis, due to the continuity of f(w) on [a,b], ∃c∈[a,b] such that f(c)=0; so w=g(w) has at least one solution. The proof is done. □

**Remark** **A1.**
*The above proof of existence relies on the Intermediate Value Theorem, which is a one-dimensional manifestation of a more general topological principle. In fact, Brouwer’s Fixed-Point Theorem states that any continuous function mapping a compact convex set into itself must have at least one fixed point. Since the closed interval [a,b] is compact and convex, and g(w) is continuous with g([a,b])⊆[a,b], the existence of a fixed point is guaranteed from this broader topological perspective.*

**Theorem** **A2** (uniqueness of fixed point). *Let g(w) and $g^{\prime }\left(w\right)$ be continuous on a real-valued on closed interval [a,b], and $k=\underset{a\leq w\leq b}{max|g^{\prime }\left(w\right)|}<1$. Then w=g(w) has a unique solution in [a,b]; i.e., g(w) has a unique fixed point $w_{n}$ in [a,b].*

**Proof.** By the Lagrange Mean Value theorem, ∀m,n such that [m,n]⊆[a,b], ∃c∈[m,n] such that $g\left(m\right)-g\left(n\right)=g^{\prime }\left(c\right)\left(m-n\right)$, so $\left|g\left(m\right)-g\left(n\right)|=\right|g^{\prime }\left(c\right)\left(m-n\right)|$ and furthermore |g(m)−g(n)|≤k|(m−n)|. Suppose that the function w=g(w) has two distinct solutions, α and β, so we have |α−β|=|g(α)−g(β)|. Such α and β satisfy α,β∈[a,b] and by the derivation above, |α−β|=|g(α)−g(β)|≤k|α−β|. This indicates that k≥1, which is a contradiction to the statement of k<1. The proof is done. □

Since 0<r<1, given an interval such that all input vectors satisfying x∈[a,b], we can verify that: (1) the equation of competitive learning rule g(w)=w+r·(x−w) is continuous on [a,b]; (2) since $g^{\prime }\left(w\right)=1-r>0$ and is a constant, the iterative function is monotonically increasing. Meanwhile, g(a)=a+r·(x−a)≥a and g(b)=b+r·(x−b)≤b. Therefore, g(w) is bounded on [a,b]×[a,b]; (3) $|g^{\prime }\left(w\right)|=|1-r|<1$. Based on the three facts, we can conclude that g(w)=w has a unique solution in [a,b], where [a,b] can be selected as any real value in the domain. We next derive the convergence of the competitive learning rule.

**Theorem** **A3** (Convergence of the competitive learning rule). *With the iterative function $w_{i+1}=w_{i}+r\cdot \left(x-w_{i}\right)$ that satisfies all the conditions in the theorems above, the weight vector $w_{i}$ linearly converges to the fixed point $w_{n}$, as well as to x.*

**Proof.** Combining equations in (A1), we have

(A2) $$\;|w_{i+1}-w_{n}|=|g\left(w_{i}\right)-g\left(w_{n}\right)|\;$$

The Taylor Expansion of $g(w_{i})$ at $w_{n}$ is $g\left(w_{i}\right)=g\left(w_{n}\right)+g^{\prime }\left(w_{n}\right)\left(w_{i}-w_{n}\right)+\frac{g^{\prime \prime }\left(w_{n}\right){\left(w_{i}-w_{n}\right)}^{2}}{2!}+\frac{g^{\prime \prime \prime }\left(w_{n}\right){\left(w_{i}-w_{n}\right)}^{3}}{3!}+\ldots$, plugging in Equation (A2) we get

(A3) $$\;|\epsilon_{i+1}\left|=\right|g^{\prime }\left(w_{n}\right)\epsilon_{i}+\frac{g^{\prime \prime }\left(w_{n}\right)\epsilon_{i}^{2}}{2!}+\frac{g^{\prime \prime \prime }\left(w_{n}\right)\epsilon_{i}^{3}}{3!}+\ldots |\;$$

where the error ϵ is denoted as $\epsilon_{i}=w_{i}-w$. When i→∞, we get

(A4) $$\;\lim_{i\to \infty }|\epsilon_{i+1}\left|=\right|g^{\prime }\left(w_{n}\right)\epsilon_{i}|\;$$

by abandoning higher-order remainders. Since $g^{\prime }\left(w\right)=1-r<1$ and $g^{\prime \prime }\left(w\right)=0$ for ∀w, the linear convergence of $|\epsilon_{i+1}|$ to zero is guaranteed as $|g^{\prime }\left(w_{n}\right)|$ exponentially accumulated. Therefore, we conclude that $w_{i}$ converges to $w_{n}$. And $w_{n}$ satisfies that $w_{n}=w_{n}+r\cdot \left(x-w_{n}\right)$, so $w_{n}=x$. This is equivalent to $w_{i}\approx x$. In addition, a bigger *r* leads to faster convergence. The proof is done. □

The entire proof only considers the dynamics of a single initial weight vector, not a batch of them. In the above derivation, we assume that the data point *x* remains fixed across updating steps. However, during the actual training process of LI-C, the input vector *x* in the update function w+r·(x−w) changes at every iteration, as the algorithm sequentially loops over all input vectors during one training epoch. This introduces a key discrepancy: while the proof provides a simplified and idealized view that facilitates tractable analysis, the real training process exhibits a richer stochastic behavior due to the continual variation in *x*, which may influence the overall convergence dynamics.

Vectorized convergence. To more precisely reflect the learning process, we vectorize the linear system of the updating steps to reflect such a dynamic. Suppose in the ${\left(i+1\right)}_{th}$ epoch, the input points that fire the same weight vector are ordered as $x_{1},x_{2},x_{3},\ldots ,x_{n}$ (the same input set is assumed to remain constant, but the order of each data point in it is shuffled for every epoch to achieve higher randomness) and the weight vectors are denoted as $w_{n}^{i+1}$, where *n* corresponds to the index of the input data point. The one-epoch training (i.e., the weight vector traversing all input samples) is outlined below:

(A5) $$\begin{array}{cc} \; & w_{1}^{i+1}=w_{0}^{i+1}+r\cdot \left(x_{1}-w_{0}^{i+1}\right)\\ \; & w_{2}^{i+1}=w_{1}^{i+1}+r\cdot \left(x_{2}-w_{1}^{i+1}\right)\\ \; & w_{3}^{i+1}=w_{2}^{i+1}+r\cdot \left(x_{3}-w_{2}^{i+1}\right)\\ \; & \ldots \\ \; & w_{n}^{i+1}=w_{n-1}^{i+1}+r\cdot \left(x_{n}-w_{n-1}^{i+1}\right) \end{array}$$

where $w_{0}^{i+1}$ is the ending vector of the previous epoch. The linear system (A5) can be vectorized and re-written as:

(A6) $$\;W_{\mathrm{new}}^{i+1}=W_{\mathrm{old}}^{i+1}+r\cdot \left(X-W_{\mathrm{old}}^{i+1}\right)\;$$

$$W_{\mathrm{new}}^{i+1}={\left[\begin{array}{c} w_{1}^{i+1}\\ w_{2}^{i+1}\\ w_{3}^{i+1}\\ \ldots \\ w_{n}^{i+1} \end{array}\right]}_{pn\times 1}\;W_{\mathrm{old}}^{i+1}={\left[\begin{array}{c} w_{0}^{i+1}\\ w_{1}^{i+1}\\ w_{2}^{i+1}\\ \ldots \\ w_{n-1}^{i+1} \end{array}\right]}_{pn\times 1}\;X={\left[\begin{array}{c} x_{1}\\ x_{2}\\ x_{3}\\ \ldots \\ x_{n} \end{array}\right]}_{pn\times 1}$$

Once the initial value of the weight vector (say, *p*-dimensional) is pre-specified, the algorithm automatically proceeds as the other elements of $W_{\mathrm{new}}^{i+1}$ and $W_{\mathrm{old}}^{i+1}$ can be computed through the epoch. During the fixed-point iterations, $W_{\mathrm{new}}^{i+1}$ at the present epoch directly acts as $W_{\mathrm{old}}^{i+2}$ (without being normalized) for the next epoch. Since Equation (A6) is in the form of a competitive learning rule with compacted vectors, the existence, uniqueness, and convergence properties still apply. In the end, we have $W_{\mathrm{new}}^{i+1}$ converging to X, and thus the mean of $W_{\mathrm{new}}^{i+1}$ approaches $\bar{X}$. Geometrically, the mean of X represents the centroid of the data chunk. That is, although the order of data points varies across training epochs—causing the large target vectors to change accordingly—their mean remains unchanged. Therefore, the averaged weight vector, through all the updating steps (in the final learning epoch), points to the center of the input data involved.

Robustness of convergence. In ideal situations with perfectly separable data, one weight vector is always associated with a fixed set of input data.In practice, due to the ambiguity inherent in the winner-take-all mechanism—especially under noisy and high-dimensional data—the same dataset may result in different sets of weight vectors being selected as “winners” across training epochs. This variability can create an impression that the smooth, deterministic nature of fixed-point convergence is disrupted. In particular, during winner selection with noisy or fluctuating inputs, the subset of input vectors $x_{1},x_{2},x_{3},\ldots ,x_{n}$ associated with a particular weight vector may change slightly over time. As a result, the effective targeting vector *X* used in the update rule may no longer remain constant across iterations, which seemingly breaks the assumptions required for classic fixed-point convergence. However, it is important to emphasize that our experimental results show that this noise-induced instability is transient. Over a sufficient number of training epochs, the dynamics exhibit a robust form of convergence: despite temporary fluctuations in the inputs or winner sets, the weight vectors consistently stabilize. This suggests that the learning process inherently absorbs such perturbations and still drives the system toward a stable configuration.

### Appendix A.3. Computational Reasoning for the Existence of Acute Partitions

The acute-partition finding procedure introduced in Algorithm 3 provides a constructive heuristic for identifying a selection of antipodal vectors whose mean forms an acute angle with every selected vector. The purpose of this subsection is not to establish a general existence theorem for arbitrary input sets, but to explain the computational reasoning underlying the heuristic and to illustrate its observed behavior through simulation.

Recall that a candidate partition is represented by

$$\tilde{X}={\left\{{\tilde{x}}_{i}\right\}}_{i=1}^{n},$$

where each ${\tilde{x}}_{i}$ is selected from the antipodal pair $\left\{x_{i},-x_{i}\right\}$. At each replacement step, the current mean is computed as

$$m=\frac{1}{n}\sum_{i=1}^{n}{\tilde{x}}_{i}.$$

The algorithm then identifies the subset

$$X_{1}=\left\{{\tilde{x}}_{i}\in \tilde{X}:{\tilde{x}}_{i}^{T}m\leq 0\right\},$$

which contains the selected vectors that do not form acute angles with the current mean. Their orientations are reversed according to

$$\tilde{X}\leftarrow \left(\tilde{X}\setminus X_{1}\right)\cup \left(-X_{1}\right).$$

This replacement step can be interpreted as an attempt to improve the directional consistency of the selected vectors. Vectors that oppose, or are orthogonal to, the current mean are replaced by their antipodal counterparts, after which the mean is recomputed. Because the mean depends on the complete selected set, reversing these vectors may also change the angular relationships of vectors that were previously acute to the mean. The procedure therefore continues iteratively until the selected set becomes stable under the replacement rule.

A stable configuration is reached when

$${\tilde{x}}_{i}^{T}m>0\;\mathrm{for}\;\mathrm{all}\;{\tilde{x}}_{i}\in \tilde{X}.$$

At this point, every selected vector forms an acute angle with the mean, and the resulting set satisfies the definition of an acute partition. In this sense, Algorithm 3 searches computationally for a stable antipodal selection rather than evaluating all possible selections explicitly.

This reasoning does not by itself guarantee convergence for every possible dataset or initialization. In particular, the update may pass through several different antipodal selections before reaching a stable configuration, and the resulting acute partition need not be unique. Nevertheless, the replacement rule provides a direct mechanism for reducing non-acute relationships relative to the current mean, offering an algorithmic explanation for why acute partitions are repeatedly obtained in our computational experiments.

To examine this behavior empirically, we apply Algorithm 3 to an artificial dataset designed to be approximately evenly distributed across the input space, thereby providing a comparatively challenging setting in which no single direction is strongly dominant. At each replacement step, we calculate the percentage of selected vectors that form acute angles with the current mean:

$$\mathrm{AcutePercentage}=\frac{\left|\left\{{\tilde{x}}_{i}\in \tilde{X}:{\tilde{x}}_{i}^{T}m>0\right\}\right|}{n}\times 100\%.$$

As shown in Figure A1, this percentage increases during the replacement process and eventually remains at 100%. This indicates that the selected vectors have reached a stable acute partition for the illustrated simulation.

Taken together, the replacement dynamics and the simulation results provide computational support for the practical existence of acute partitions under the conditions examined in this work. This evidence should be interpreted as reasoning in support of the heuristic behavior of Algorithm 3, rather than as a rigorous proof that the procedure must converge for every arbitrary real-valued input set.

### Appendix A.4. Reasoning for Single-Neuron Convergence to an Acute-Partition Mean

This subsection provides additional algorithmic reasoning for Proposition 1. The purpose is to explain why, once the antipodal selection associated with a single neural weight vector becomes stable, repeated AIME updates drive that weight vector toward the mean of the corresponding acute partition.

Consider a single neural weight vector *w* trained on the input set *X*. For each input vector $x_{i}$, the AIME update rule selects either $x_{i}$ or $-x_{i}$ according to its orientation relative to the current weight vector. The resulting selected set is denoted by

$$\tilde{X}={\left\{{\tilde{x}}_{i}\right\}}_{i=1}^{n}.$$

Within one training epoch, the weight vector is updated sequentially toward the vectors in $\tilde{X}$. For a sufficiently small learning rate, each individual update produces only a limited change in direction. Consequently, the antipodal selection typically changes gradually rather than discontinuously across successive updates.

If the selected set $\tilde{X}$ remains fixed during an interval of training, the update dynamics reduce to repeated movement of the weight vector toward the vectors in that fixed set. Averaged over a complete epoch, these updates move the weight vector toward the mean

$$m=\frac{1}{n}\sum_{i=1}^{n}{\tilde{x}}_{i}.$$

Thus, for a stable antipodal selection, the mean vector *m* acts as the effective target of the single-neuron learning dynamics.

During early training, the selected set may change because updates to *w* can alter the signs of some inner products $w^{T}x_{i}$. Each such change replaces one selected vector by its antipodal counterpart and therefore produces a new candidate partition and a new mean. The weight vector and its associated antipodal selection are consequently updated together. This process continues until the directional changes in *w* are sufficiently small that the selected set no longer changes under successive epochs.

At such a stable configuration, every selected vector satisfies

$${\tilde{x}}_{i}^{T}m>0,$$

so the selected set forms an acute partition and *m* is its acute-partition mean. Repeated updates then keep the weight vector in the neighborhood of this mean. Small residual fluctuations may remain because the inputs are presented sequentially, but these fluctuations do not alter the limiting interpretation provided that they do not change the stable antipodal selection.

This reasoning supports the convergence behavior summarized in Proposition 1: once the antipodal selection becomes stable, a single AIME-trained neural weight vector approaches the mean of the corresponding acute partition. The argument is intended as an algorithmic interpretation of the observed convergence dynamics rather than as a general closed-form proof for every possible dataset, initialization, and learning-rate schedule.

### Appendix A.5. Reasoning for Preferential Convergence Toward the SAPM

This subsection provides additional reasoning for Proposition 2. Rather than establishing a rigorous proof, the following discussion explains why the AIME dynamics tend to favor convergence toward the strongest acute-partition mean (SAPM) once multiple acute partitions are available.

As discussed in the previous subsection, a single neural weight vector converges toward the mean of a stable acute partition. However, when several acute partitions exist, not all of them are equally stable under the AIME update rule. Their stability depends on the spatial concentration of the selected vectors around their corresponding partition means.

Consider two candidate acute partitions. In one partition, the selected vectors are tightly concentrated around their mean, while in the other, the selected vectors are more broadly distributed and several vectors lie close to the partition boundary. Small perturbations of the weight vector therefore have very different consequences. In the tightly concentrated partition, slight changes in the weight vector do not alter the antipodal selection, so the partition remains unchanged. By contrast, in the more weakly concentrated partition, small directional changes may cause one or more vectors to switch to their antipodal counterparts, thereby generating a different partition and a different target mean.

Figure A2b illustrates this intuition. The blue region represents a weaker acute partition. Because several vectors lie close to the decision boundary, a small perturbation of the weight vector may reverse the selection of vectors such as $x_{2}$ and $x_{3}$, producing a different antipodal selection and consequently a different partition. In contrast, the yellow region represents a more concentrated acute partition. The selected vectors remain acute under small perturbations, so the corresponding partition is preserved. Consequently, the weight vector is more likely to remain in the neighborhood of this partition throughout subsequent training.

This observation suggests that acute partitions exhibiting stronger directional concentration possess greater stability under the iterative dynamics of AIME. We therefore refer to the corresponding partition mean as the strongest acute-partition mean (SAPM). The SAPM should be interpreted as a computational characterization of the empirically preferred convergence point, rather than as a quantity derived through a closed-form mathematical optimization.

Taken together with the simulation results presented in the main text, this reasoning explains why independently initialized neural weight vectors repeatedly converge toward the SAPM in practice, thereby supporting the preferential convergence behavior summarized in Proposition 2.
